# Characterization of microbial associations with methanotrophic archaea and sulfate-reducing bacteria through statistical comparison of nested Magneto-FISH enrichments

**DOI:** 10.7717/peerj.1913

**Published:** 2016-04-18

**Authors:** Elizabeth Trembath-Reichert, David H. Case, Victoria J. Orphan

**Affiliations:** Department of Geological and Planetary Sciences, California Institute of Technology, Pasadena, CA, United States

**Keywords:** Magneto-FISH, Co-occurrence network, Earth microbiome project, iTag sequencing, Anaerobic oxidation of methane, Microbial association, Microbial ecology

## Abstract

Methane seep systems along continental margins host diverse and dynamic microbial assemblages, sustained in large part through the microbially mediated process of sulfate-coupled Anaerobic Oxidation of Methane (AOM). This methanotrophic metabolism has been linked to consortia of anaerobic methane-oxidizing archaea (ANME) and sulfate-reducing bacteria (SRB). These two groups are the focus of numerous studies; however, less is known about the wide diversity of other seep associated microorganisms. We selected a hierarchical set of FISH probes targeting a range of *Deltaproteobacteria* diversity. Using the Magneto-FISH enrichment technique, we then magnetically captured CARD-FISH hybridized cells and their physically associated microorganisms from a methane seep sediment incubation. DNA from nested Magneto-FISH experiments was analyzed using Illumina tag 16S rRNA gene sequencing (iTag). Enrichment success and potential bias with iTag was evaluated in the context of full-length 16S rRNA gene clone libraries, CARD-FISH, functional gene clone libraries, and iTag mock communities. We determined commonly used Earth Microbiome Project (EMP) iTAG primers introduced bias in some common methane seep microbial taxa that reduced the ability to directly compare OTU relative abundances within a sample, but comparison of relative abundances between samples (in nearly all cases) and whole community-based analyses were robust. The iTag dataset was subjected to statistical co-occurrence measures of the most abundant OTUs to determine which taxa in this dataset were most correlated across all samples. Many non-canonical microbial partnerships were statistically significant in our co-occurrence network analysis, most of which were not recovered with conventional clone library sequencing, demonstrating the utility of combining Magneto-FISH and iTag sequencing methods for hypothesis generation of associations within complex microbial communities. Network analysis pointed to many co-occurrences containing putatively heterotrophic, candidate phyla such as OD1, *Atribacteria*, MBG-B, and Hyd24-12 and the potential for complex sulfur cycling involving *Epsilon*-, *Delta*-, and *Gammaproteobacteria* in methane seep ecosystems.

## Introduction

A central goal in microbial ecology is identifying and understanding microbial interactions in the environment. This goal can be addressed at many scales from statistical analyses of entire ecosystems (Barberán et al., 2012; Malfatti & Azam, 2010; Ruff et al., 2015; Steele et al., 2011; Sunagawa et al., 2015) to high resolution image analysis of specific symbioses (Malfatti & Azam, 2010; McGlynn et al., 2015; Orphan, 2009; Orphan et al., 2001b; Wegener et al., 2015). Previous studies have shown that complex datasets can be distilled to determine primary ecosystem drivers, such as temperature, as main predictors of community variability (Sunagawa et al., 2015). In addition to correlating microbial patterns to environmental factors, interspecies interactions can be evaluated with methods such as co-occurrence analysis (Friedman & Alm, 2012). Statistical significance of co-occurrence can be assessed at scales ranging from the entire genome to the operational taxonomic unit (OTU) (Barberán et al., 2012; Chaffron et al., 2010).

Many physical separation methods have been developed to partition complex microbial assemblages before analysis, including fluorescence-activated flow sorting (Amann et al., 1990; Yilmaz et al., 2010), optical trapping (Ashkin, 1997), microfluidics (Melin & Quake, 2007), and immunomagnetic beads (Pernthaler et al., 2008; Šafařík & Šafaříková, 1999) that use characteristics of interest such as phylogenetic identity (Fluorescence *In Situ* Hybridization; FISH) or activity (Hatzenpichler & Orphan, 2015; Hatzenpichler et al., 2014; Kalyuzhnaya, Lidstrom & Chistoserdova, 2008; Wegener et al., 2012; Berry et al., 2015).

Here we combine Magneto-FISH and Illumina Tag (iTag) sequencing utilizing the Earth Microbiome Project (EMP) universal primer set (Caporaso et al., 2012). The Magneto-FISH method was originally developed to enrich for and characterize multi-species microbial associations in environmental samples (Pernthaler et al., 2008). This method consists of a liquid CARD (CAtalyzed Reporter Deposition)-FISH reaction as a 16S rRNA gene identity-based selection mechanism followed by an immunomagnetic sediment matrix separation mechanism to target specific phylogenetic groups in conjunction with their physically associated microbial partners. By combining this method for phylogenetically targeted physical separation with high throughput amplicon sequencing, we can compare an array of associated microbial communities in parallel, with replicates. This provides statistical power in deriving microbial associations from complex sediment community assemblages, and thereby improving hypothesis development.

Anaerobic methane-oxidizing (ANME) archaea and sulfate-reducing *Deltaproteobacteria* (SRB) are the predominant community members discussed in methane seep literature and form syntrophic partnerships in physical associations, termed “aggregates” or consortia (Boetius et al., 2000; Green-Saxena et al., 2014; Knittel et al., 2003; Orphan et al., 2001a; Schreiber et al., 2010). Since physical association appears to be an important element for consortia activity (McGlynn et al., 2015; Wegener et al., 2015), methods like Magneto-FISH are ideal for probing this system because target organisms are separated from the sediment matrix along with their physically associated partners. A hierarchical probe set was chosen targeting *Deltaproteobacteria* and their ANME partners to create nested Magneto-FISH enrichments from methane seep sediment incubations under methane headspace. This method allows us to examine potential physical associations between ANME and SRB taxa and other microorganisms using co-occurrence statistical methods applied to iTag sequences from nested Magneto-FISH enrichments.

ANME have been broadly divided into three separate groups, which can be further subdivided into ANME-1a, 1b, 2a, 2b, 2c, and 2d, and 3. ANME-1 archaea are a unique order-level lineage within the *Euryarchaeota*, between the *Methanomicrobiales* and the *Methanosarcinales*, known to associate with sulfate-reducing bacteria, but obligately associated lineages have yet to be defined. ANME-2 archaea, within the order *Methanosarcinales*, commonly form associations with *Desulfosarcina*/*Desulfococcus*-related (DSS) sulfate-reducing *Deltaproteobacteria* (Boetius et al., 2000; Orphan et al., 2001a; Schreiber et al., 2010). They have also been found in association with *Desulfobulbus*-related (DSB) *Deltaproteobacteria* in the same environments, where geochemical factors have been suggested as a possible explanation for partner differentiation (Green-Saxena et al., 2014). ANME-2a/b and ANME-2c both predominately associate with a subgroup of DSS, SEEP-SRB1 (Schreiber et al., 2010), but also form consortia with DSB (Green-Saxena et al., 2014; Pernthaler et al., 2008). ANME-3 has been found in association with *Desulfobulbus*-related *Deltaproteobacteria* (Niemann et al., 2006) and SEEP-SRB1 (Schreiber et al., 2010). These ANME groups have also been observed in the environment without bacterial partners (House et al., 2009; Orphan et al., 2002; Schreiber et al., 2010; Treude et al., 2007). In addition to ANME archaea, other uncultured archaeal lineages commonly recovered from methane seeps include Marine Benthic Group-D (*Thermoplasmatales*), Deep Sea Archaeal Group/Marine Benthic Group-B (Ruff et al., 2015; Yanagawa et al., 2011), and sometimes methanogens (Orphan et al., 2001a; Ruff et al., 2015; Takano et al., 2013; Vigneron et al., 2015).

*Deltaproteobacteria* diversity beyond DSS and DSB has also been well described in methane seeps. In addition to SEEP-SRB1, Knittel et al. (2003) define three more *Deltaproteobacteria* clades within *Desulfobulbaceae* (SEEP-SRB2, 3 and 4). Green-Saxena et al. (2014) also described a *Desulfobulbaceae* affiliated seepDBB group in methane seep systems. Bacterial diversity surveys of methane seep habitats frequently report occurrence of other diverse *Proteobacteria* including sulfur oxidizers (*Gammaproteobacteria* and *Epsilonproteobacteria*) and putative heterotrophs (*Alphaproteobacteria* and *Betaproteobacteria*) (Pernthaler et al., 2008; Ravenschlag et al., 1999). Many other bacterial phyla have also been found such as *Firmicutes*, *Thermomicrobia*, *Bacteroidetes*, *Chlorobi*, *Nitrospira*, WS3, OD1, OP11, TM7, and WS6 (Schreiber et al., 2010); *Cytophaga* and *Flavobacteria* (Knittel et al., 2003); *Chloroflexi*, *Atribacteria* (previously Candidate Division JS1), CD12, WS1, OS-K, AC1, and *Planctomycetes* (Yanagawa et al., 2011); and *Acidobacteria* (Ravenschlag et al., 1999). Ruff et al. (2015) identified *Methanomicrobia*, *Deltaproteobacteria*, Hyd24-12 and Atribacteria (JS1) as the characteristic ‘core’ microbial taxa in methane seep ecosystems, as compared to *Gammaproteobacteria*, *Flavobacteria*, *Thermoplasmatales*, and MBG-B taxa that were found in high relative abundance in seeps and other marine ecosystems.

Despite the wealth of bacterial and archaeal diversity in methane seep sediments, little is known about potential associations with ANME/SRB, or associations that do not involve ANME or SRB. Our study utilizes the novel combination of targeted Magneto-FISH enrichment of specific microbial taxonomic groups and iTag sequencing to develop statistically supported co-occurrence microbial networks to address knowledge gaps in our understanding of methane seep microbial communities. Network analysis revealed many novel associations between methane seep *Proteobacteria* taxa and Candidate phyla. The significant co-occurrence observed by these OTUs suggests new avenues for future studies on microbial interactions involved in carbon and sulfur cycling in methane seep systems.

## Materials & Methods

### Sample collection and Magneto-FISH

iTag Magneto-FISH enrichments were conducted using a large scale (1 L) incubation of methane seep sediment from Hydrate Ridge North (offshore Oregon, USA) collected in September 2011 at 44°40.0${2}^{^{\prime}}\mathrm{N}$ 125°6.0${0}^{^{\prime}}\mathrm{W}$, from a water depth of 775 m using the ROV *JASON* II and the R/V *Atlantis*. Marine sediment was collected using a push core to sample a sulfide-oxidizing microbial mat adjacent to an actively bubbling methane vent. A sediment slurry from the upper 0–15 cm depth horizon of the push core was prepared with 1 volume N_2_ sparged artificial seawater to 1 volume sediment, overpressurized with methane (3 bar) and incubated at 8 °C in a 1 L Pyrex bottle capped with a butyl rubber stopper until subsampling for Magneto-FISH.

In February 2015, incubation samples were immediately fixed in 0.5 ml sediment aliquots in 2% paraformaldehyde (PFA) for 3 h at 4 °C. The samples were washed in 50% phosphate-buffered saline (PBS): 50% EtOH, then 75% EtOH: 25% DI water, and resuspended in 2 volumes (1 ml) 100% ethanol. Samples were centrifuged at 1,000 × *g* for 1 min between wash steps. After fixation, the Magneto-FISH method first described by Pernthaler et al. (2008) and further optimized by Schattenhofer & Wendeberg (2011) and Trembath-Reichert, Green-Saxena & Orphan (2013) was used. Briefly, a liquid CARD-FISH reaction was followed by immunomagnetic bead incubation coupled with anti-fluorescein attaching magnetic beads to CARD-FISH hybridized aggregates. Samples were then held against magnets and the sediment matrix was washed away, retaining target cells and physically associated microbes in the magnetic portion as described in Trembath-Reichert, Green-Saxena & Orphan (2013). Four previously published FISH probes were used targeting a range of *Deltaproteobacteria* and *Methanomicrobia* (Table 1). A subset of three 0.5 ml aliquots was also immediately frozen before fixation (unfixed bulk sediment), and another three aliquots were frozen after fixation (fixed bulk sediment) for bulk sediment comparison with Magneto-FISH enrichments. Sediment for MSMX-Eel_932 Magneto-FISH metabolic gene analysis was fixed and washed onboard in September 2011, as described above. See methods flow chart provided in Fig. S1.

**Table 1:** FISH probes and PCR primers used in this studys. FISH probes for Magneto-FISH and CARD-FISH and PCR primers for iTag and Clone gene libraries with oligonucleotide sequence, target organisms, references, and formamide concentration (FISH) or annealing temperature (PCR).

Name	Sequence (5′ → 3′)	Target	Reference	FA (%)/ Annealing (°C)
**PROBES for Magneto-FISH & CARD-FISH **				
DSS_658	TCCACTTCCCTCTCCCAT	*Desulfosarcina/Desulfococcus*, Desulfofaba, Desulfofrigus	Manz et al. (1998)	50
Delta_495a	AGTTAGCCGGTGCTTCCT	Most *Deltaproteobacteria* and most Gemmatimonadetes	Loy et al. (2002)	35
Delta_495a-comp	AGTTAGCCGGTGCTTCTT		Loy et al. (2002)	35
Seep-1a_1441	CCCCTTGCGGGTTGGTCC	Seep-SRB1a	Schreiber et al. (2010)	45
MSMX-Eel_932	AGCTCCACCCGTTGTAGT	All ANME groups	Boetius et al. (2000)	35
ANME-1_350	AGTTTTCGCGCCTGATGC	ANME-1	Boetius et al. (2000)	40
Epsi_404	AAAKGYGTCATCCTCCA	*Epsilonproteobacteria*	Macalady et al. (2006)	30
Gam_42a	GCCTTCCCACATCGTTT	*Gammaproteobacteria*	Manz et al. (1992)	35
Gam_42a comp (Bet42a)	GCCTTCCCACTTCGTTT	*Betaproteobacteria*	Manz et al. (1992)	35
Pla_46	GACTTGCATGCCTAATCC	*Planctomycetes*	Neef et al. (1998)	35
Pla_886	GCCTTGCGACCATACTCCC	*Planctomycetes*	Neef et al. (1998)	35
CF_319A	TGGTCCGTGTCTCAGTAC	CFB (*Cytophaga, Bacteriodales, Flavobacterium, Sphingobacterium*)	Manz et al. (1996)	35
CF_319B	TGGTCCGTATCTCAGTAC	CFB (mostly *Cytophaga*)	Manz et al. (1996)	35
**PRIMERS for iTAG**				
515F	GTGCCAGCMGCCGCGGTAA	V4 region universal 16S rRNA	Caporaso et al. (2012)	55
806R	GGACTACHVGGGTWTCTAAT	V4 region universal 16S rRNA	Caporaso et al. (2012)	55
**PRIMERS for CLONE LIBRARIES**				
Bac27F	AGAGTTTGATYMTGGCTC	Bacterial 16S rRNA	Lane (1991)	54
U1492R	GGYTACCTTGTTACGACTT	Universal 16S rRNA	Lane (1991)	54
10-30Fa	TCCGGTTGATCCTGCC	Archaeal 16S	Von Wintzingerode et al. (1999)	54
Arc958R	YCCGGCGTTGAMTCCAATT	Archaeal 16S	DeLong (1992)	54
DSR1F	ACSCACTGGAAGCACG	dsrAB	Wagner et al. (1998)	Touchdown 61-48
DSR4R	GTGTAGCAGTTACCGCA	dsrAB	Wagner et al. (1998)	Touchdown 61-48
APS_1F	TGGCAGATCATGATYMAYGG	APS reductase	Blazejak et al. (2006)	54
APS_4R	GCGCCAACYGGRCCRTA	APS reductase	Blazejak et al. (2006)	54
sox527F	TGGTWGGWCAYTGGGAATTTA	sulfate thiol esterase	Akerman, Butterfield & Huber (2013)	46
sox1198R	AGAANGTATCTCKYTTATAAAG	sulfate thiol esterase	Akerman, Butterfield & Huber (2013)	46

### iTag amplification

For iTag sequencing, ten Magneto-FISH enrichments were performed in parallel using the FISH probes DSS_658 (triplicate), MSMX-Eel_932 (triplicate), SEEP-1a_1441 (duplicate), Delta_495a + Delta_495a competitor (duplicate). Magneto-FISH enrichments and bulk sediment samples were resuspended in 650 µl solution PM1 and transferred to silica tubes from the PowerMicrobiome RNA Isolation Kit (MoBio). This kit was chosen based on manufacturer recommendation for formalin-fixed sediment samples, with the added capability to co-elute RNA if desired. 6.5 µl of beta-mercaptethanol was added, and samples were mechanically lysed in a bead beater (FastPrepFP120; Thermo Electron Corp., Waltham, MA, USA) for 45 s at setting 5.5 and incubated at 65 °C for 3.5 h. The remaining steps in the PowerMicrobiome RNA Isolation Kit were followed according to manufacturer instructions (starting at step 5) without any DNase procedures, and eluting in a final volume of 60 µl ultrapure water. DNA extracts were quantified using a Qubit Flurometer and HS dsDNA kit (Invitrogen; Table S1). All but one Magneto-FISH sample had DNA concentrations below detection (<0.5 ng/µl); however, all samples yielded PCR amplicons when viewed on a gel after initial pre-barcoding PCR (30 cycles).

**Table 2:** 16S rRNA gene iTag and clone library relative sequence abundance for seep microbiome OTUs. Relative sequence abundances were computed for the top 135 OTUs in the iTag dataset. These OTUs correspond to ∼55% of the total sequences in the unfixed bulk sediment. Bacterial and archaeal 16S rRNA gene libraries are included for the core methane seep taxa, with the total number of clones for each library indicated above. Core methane seep taxa were based on Ruff et al. (2015) and include: candidate Phylum *Atribacteria*, Candidate Division Hyd24-12, *Methanomicrobia*, *Caldilineales*, *Desulfobacterales*, and *Spirochaetales*. While we did recover other *Chloroflexi*, no *Caldilineales* were recovered in iTag or gene library sequencing so they are not included below. Fixed bulk sediment was chosen for baseline comparison (rather than unfixed) since it includes the potential loss of cells due to fixation and wash steps, thereby processed more similarly to the Magneto-FISH samples. An average and standard deviation for relative sequence abundance among replicates was calculated for each sample set. A ratio of the average relative sequence abundance of Magneto-FISH enrichments compared to the fixed bulk sediment value is reported (Rel. fixed). Ratios over 1.5 are underlined. 16S rRNA gene bacteria and archaea clone libraries for two Magneto-FISH enrichments and fixed bulk sediment are also included for comparison to recovered iTag diversity.

	16S rRNA gene (iTAG)																16S rRNA gene (Clone Library)				
	Seep1a_1441			DSS_658			Delta_495a			MSMX-Eel_932			Fixed bulk		Unfixed bulk		DSS_658		MSMX-Eel_932		Fixed bulk
Taxon	Avg.	Stdev.	Rel. fixed	Avg.	Stdev.	Rel. fixed	Avg.	Stdev.	Rel. fixed	Avg.	Stdev.	Rel. fixed	Avg.	Stdev.	Avg.	Stdev.	24 arc, 41 bac	Rel. fixed	60 arc, 87 bac	Rel. fixed	43 arc, 95 bac
*ANME-1a*	0.07	0.07	0.67	0.04	0.04	0.36		0.01	0.05	0.07	0.01	0.61	0.11	0.02	0.10	0.02	0.08	0.28	0.08	0.28	0.30
*ANME-1b*	0.11	0.08	0.92	0.09	0.05	0.74	0.12	0.05	0.95	0.15	0.09	1.22	0.12	0.03	0.08	0.01					0.14
*ANME-2a/b*		0.01	0.19	0.01	0.01	0.31			0.01			0.11	0.02	0.01	0.01		0.42	2.24	0.47	2.51	0.19
*ANME-2c*			0.01			0.09							0.01	0.01	0.01		0.50	1.54	0.42	1.28	0.33
*Desulfobacula*																			0.01		
*Desulfobulbus*	0.08	0.06	1.01	0.11	0.05	1.30	0.20	0.14	2.52	0.03	0.01	0.36	0.08	0.01	0.12	0.01	0.05	0.66	0.06	0.78	0.07
*Desulfocapsa*	0.02	0.01	1.02			0.16		0.01	0.32	0.02	0.03	1.10	0.01		0.01		0.05				
*Desulfococcus*	0.03	0.03	0.67	0.03	0.03	0.61	0.03	0.04	0.74	0.08	0.13	1.85	0.04		0.03				0.08	3.82	0.02
*Desulfoluna*	0.01	0.02	2.20	0.02	0.02	4.62				0.04	0.04	8.47	0.01		0.01						
*SEEP-SRB1*	0.13	0.07	2.36	0.05	0.01	0.84	0.04	0.01	0.78	0.09	0.08	1.67	0.06		0.06		0.22	1.74	0.34	2.73	0.13
*SEEP-SRB2*	0.02	0.02	0.33	0.04	0.03	0.85	0.01	0.01	0.19	0.07	0.05	1.35	0.05	0.01	0.05		0.15	2.78	0.06	1.09	0.05
*SEEP-SRB4*	0.01	0.02	1.34	0.01	0.01	1.30		0.01	0.39			0.12	0.01		0.01				0.03	3.28	0.01
*Hyd24-12*	0.04	0.03	3.44	0.01	0.02	1.15			0.03	0.02	0.03	1.73	0.01		0.01						
*Atribacteria*	0.02	0.03	1.51	0.08	0.07	4.80	0.05	0.07	3.02	0.12	0.12	7.18	0.02		0.02						
*Spirochaeta*		0.01	0.76	0.02	0.03	4.36				0.01	0.01	1.63			0.01		0.02	1.16	0.03	1.64	0.02

iTag samples were prepared with Earth Microbiome Project (EMP) primers 515f and 806r (Caporaso et al., 2012). An initial amplification of 30 cycles with primers lacking the barcode, linker, pad, and adapter was performed for all samples, in duplicate. Duplicate PCR reactions were pooled and reconditioned for 5 cycles with barcoded primers, for a total of 35 cycles. A master mix of 2 X Q5 Hot Start High Fidelity Master Mix (NEB) and 10 µM forward and reverse primers was prepared for a final volume of 15 µl per sample, with 1 µl DNA template. PCRs had an initial 2 min heating step at 98 °C, followed by cycles of 10 s 98 °C, 20 s 54 °C, and 20 s 72 °C, and finished with a final extension of 2 min at 72 °C. PCR negative controls, substituting ultrapure water for DNA template, were amplified for 40 cycles total. We note that these are not the official recommended reagents or PCR conditions from the EMP, but internal lab tests showed that for 6 out of 9 mock community taxa, recovered sequence relative abundances were more accurate when using Q5 polymerase rather than the recommended Hot Start MasterMix (5-prime). EMP primers were chosen for iTag for cross-comparison between studies, though there is known primer bias within this universal primer set (Parada et al., 2015) and sequencing reactions will always have some inherent variability.

### Mock communities

Four mock communities were prepared with a range of relative proportions of nine common methane seep taxa (Table S2). Full-length 16S rRNA gene plasmids from each taxa listed were quantified by Qubit. Taking into account the plasmid’s nucleotide composition and length in order to calculate its molecular weight, plasmids were quantitatively combined in known volumetric fractions to achieve a range of desired mock community compositions. These combined plasmid mixes were diluted to ∼1 ng/µL and then prepared according to the same iTag methods as all other samples.

### iTag sequence processing

We followed the *mothur* Standard Operating Procedure (SOP) for Illumina MiSeq sequencing of the 16S rRNA gene V4 region, accessed May 2015 and using methods described in Kozich et al. (2013) with UCHIME chimera checking (Edgar et al., 2011). A concatenated file of the *mothur* version of separate archaeal and bacterial SILVA 119 databases (Quast et al., 2013) was used for alignment and classification. Unfixed Bulk Sediment 1 only returned 8% of the average DNA concentration of the other two samples. (Table S1). This sample was removed from statistical analyses because it fails to be a representative of the unfixed bulk sediment community baseline. The mock communities were processed following the “Assessing Error Rates” section of the *mothur* SOP to compute sequencing error rates and spurious OTU rates (Table S4). Additional analysis demonstrating sequence processing did not selectively remove ANME-2c sequences and relative sequence abundances recovered with iTag sequencing of mock communities are provided in Tables S3 and S2, respectively.

Using *R* version 3.1.3 (R Core Team, 2015), an average number of sequences per OTU was calculated from unfixed bulk sediment samples (2 and 3). All OTUs with an average relative sequence abundance below 0.1% in the unfixed bulk sediment were identified and removed from all samples using *mothur*. 135 unique OTUs remained out of 25,354. We also verified that after the 0.1% cutoff was applied, no negative control contaminant OTUs remained. The top 20 OTUs amplified from the no template negative control were classified as, in order of sequence abundance: Sphingomonas*; Planctomyces*; Escherichia-Shigella*; Staphylococcus; Roseomonas*; Pir4_ lineage; Delftia*; Macrococcus; Myxococcales;0319-6G20;unclassified; Planctomyces; Enhydrobacter; Sphingobium*; Caenispirillum; Bacillus*; Pseudoxanthomonas*; Peptoniphilus; Lysobacter; Salinicoccus; Propionibacterium.* Reagent contaminant genera discussed in Salter et al. (2014) are denoted by (*). All samples (including mock community and negative controls) were submitted to the SRA under the accession SAMN03879962, BioSample: SAMN03879962, Sample name: PC47 (5133-5137) mixed slurry.

Gene libraries of the Magneto-FISH samples were prepared as in Trembath-Reichert, Green-Saxena & Orphan (2013) using the primers and annealing temperatures listed in Table 1 and TOPO TA Cloning Kit for Sequencing with pCR4-TOPO Vector and One Shot Top 10 chemically competent Escherichia coli (Life Technologies). All full-length 16S rRNA gene sequences were aligned by the SINA online aligner (**v1.2.11**) (Pruesse, Peplies & Glöckner, 2012) and added using maximum parsimony to the SILVA 119 database (Quast et al., 2013) for classification. A taxonomy-based count table was prepared (sequences per taxa, per sample) and all taxa absent from the bulk sediment library were removed from Magneto-FISH enrichment libraries (for parity with iTag contaminant removal processing). Functional gene sequences were translated using the *EMBOSS* online translation tool (Li et al., 2015), then added to *ARB* (Ludwig et al., 2004) databases for phylogenetic placement and classification. Sequences were submitted to NCBI under the following accession numbers: AprA ( KT280505– KT280517), DsrA ( KT280518– KT280533), McrA ( KT280534– KT280581), Archaeal 16S rRNA gene ( KT280582– KT280632), Bacterial 16S rRNA gene ( KT280633– KT280909), SoxB ( KT280910– KT280928). Gene trees were computed with representative sequences using PhyML 3.0 (Guindon et al., 2010) online execution with defaults on the South of France Bioinformatics platform.

### Statistical analysis

**Figure 1:**
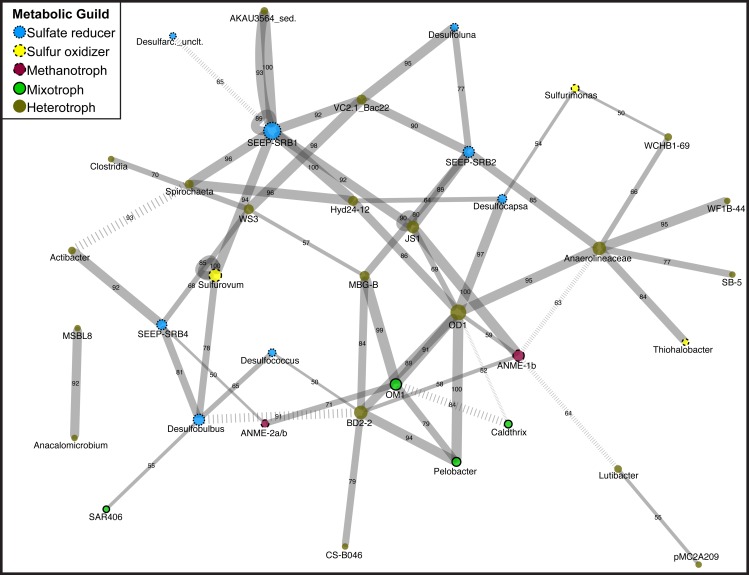
Network diagram of Magneto-FISH and bulk sediment samples. Co-occurrence analysis of the top 135 unique OTUs displayed in network form. Nodes represent the taxonomy of the OTUs in the network and edges are the connections between OTUs. Node size is scaled by number of connecting OTUs and colored by simplified, putative metabolic guild (sulfate reducer: blue, small dash; sulfur oxidizer: yellow, medium dash; archaeal methanotroph: magneta, large dash; mixotroph: green, no dash; heterotroph: brown, no outline). Edge thickness is scaled by number of occurrences of this association (from 50 to 100 times) and number of occurrences also included along edge. Negative associations are denoted by hashed lines. The combined network is displayed using Cytoscape, with the average correlation coefficient across all runs determining the distance between nodes and the number of occurrences in 100 network iterations determining edge width.

Weighted UniFrac (Lozupone & Knight, 2005), Metastats (White, Nagarajan & Pop, 2009), and linear discriminant analysis (LDA) effect size (LEfSe) (Segata et al., 2011) analyses were computed in *mothur* as outlined in the *mothur* SOP. Co-occurrence statistical analyses were run using the table of 135 unique OTUs in the format of sequence counts of each OTU per sample. The program *SparCC* was used to determine significant correlations (Friedman & Alm, 2012). This analysis was run 100 times with default settings, except 10 iterations were used instead of 20. OTUs with *SparCC* correlations above an absolute value of 0.6 with *p*-values below 0.01 were considered significant. Resulting associations that occurred in at least 50 out of 100 network iterations are provided in Table S5. *Cytoscape* (Shannon et al., 2003) was used to display associations in Fig. 1.

### CARD-FISH microscopy

A triple CARD-FISH hybridization was performed with bacterial probes listed in Table 1, ANME-1_350 and MSMX-Eel_932. The sample preparation and CARD reaction was performed as per Green-Saxena et al. (2014). After the three CARD reactions, samples were post-stained with DAPI (25 ng/µl). CARD signal within any part of a physically attached group of cells larger than 10 µm was counted as a positive identification. For example, a large EPS matrix that contained many smaller separate ANME-1 and ANME-2 aggregates would count as one positive identification for each clade. This was done to simulate groups that would have been isolated together in a Magneto-FISH enrichment. Since the MSMX-Eel_932 probe also targets the ANME-1 population, only cells with MSMX-Eel_932 signal and no ANME-1_350 signal were recorded as an ANME-2 positive identification to comprehensively target ANME-1, -2, and a bacterial partner in a triple CARD-FISH hybridization set. ANME-3 were not recovered in the iTag dataset and were not considered as potential contributors to MSMX-Eel_932 signal.

## Results

### Relative sequence abundance of seep microbiome taxa in 16S rRNA gene iTag and libraries

Relative sequence abundances of the methane seep microbiome characteristic taxa, ANME archaea, *Deltaproteobacteria*, Hyd24-12, and Atribacteria (Ruff et al., 2015), were compared two ways: (1) between iTag and gene library 16S rRNA gene samples to determine how relative sequence abundances differed between sequencing methodologies, and (2) between Magneto-FISH enrichment and bulk sediment to determine taxa-specific relative sequence abundance for each probe (Table 1).

Mock community analysis showed that ANME-2 were always underrepresented in iTag data (0.32–0.81 fold of what was expected), whereas the *Deltaproteobacteria* and ANME-1b were more faithfully represented (Table S2). ANME-1a was consistently overamplified. By normalizing the relative sequence abundance of ANME-2c, -2a/b, and -1a to the abundance of ANME-1b, the most faithfully amplified archaea in the mock community data (Table S2), we could compute a ratio between the average relative sequence abundance in fixed bulk sediment samples between iTag and the archaeal 16S rRNA gene library. ANME-2c (0.04 iTag:clone ratio), ANME-2a/b (0.12), and ANME-1a (0.40) were all less abundant in iTag sequences as compared to the archaeal gene library (calculated from values in Table 2). Similarly comparing SEEP-SRB1 to *Desulfobulbus* between the two methods in fixed bulk sediment returns a ratio of 0.41 iTag:clone. Since the iTag methodology recovers far more diversity (e.g., *Desulfobacula*, *Desulfocapsa*, *Desulfoluna*, Atribacteria, and Hyd24-12 were not recovered in the bacterial 16S rRNA gene bulk sediment library), it is expected that the relative sequence abundances of each individual taxon computed from iTag data would be less than the domain targeted 16S rRNA gene libraries. However the ANME-2c abundance ratio was an order of magnitude less than ANME-1a and SEEP-SRB1 ratios, and appears to be an extreme case of underestimation in iTag data. There was also variation between Magneto-FISH enrichment replicates, as indicated by the high standard deviations of Magneto-FISH samples as compared to bulk sediment samples. The degree of variation (average standard deviation across all taxa listed) correlated with the specificity of the probe; where Delta_495a had the lowest average standard deviation and Seep-1a_1441 had the highest average standard deviation.

The high relative sequence abundance taxa (>1.5 fold relative sequence abundance increase over fixed bulk sediment; Table 2) in the averaged Seep-1a_1441 iTag Magneto-FISH enrichments were *Desulfoluna* (2.20), SEEP-SRB1 (2.36), Hyd24-12 (3.44) and *Atribacteria* (1.51) (Table 2). The DSS_658 enrichment had fewer high relative sequence abundance taxa with only *Desulfoluna* (4.62), *Spirochaeta* (4.36), and *Atribacteria* (4.80). The Delta_495a enrichment also had three high relative sequence abundance taxa with *Desulfobulbus* (2.52), *Spirochaetae*-uncultured (3.70), and *Atribacteria* (3.02). The MSMX-Eel_932 enrichment had six high relative sequence abundance taxa with *Desulfococcus* (1.85), *Desulfoluna* (8.47), SEEP-SRB1 (1.67), *Spirochaeta* (1.63), Hyd24-12 (1.73), and *Atribacteria* (7.18). Gene library results showed high relative sequence abundance (>1.5) in both ANME and *Deltaproteobacteria* with DSS_658 and MSMX-Eel_932 enrichments (Table 2). Similar to the bulk sediment, *Desulfobacula*, *Desulfocapsa*, *Desulfoluna*, *Atribacteria* and Hyd24-12 were not recovered in the bacterial 16S rRNA gene Magneto-FISH libraries. MSMX-Eel_932 enriched for SEEP-SRB1 (2.73), SEEP-SRB4 (3.28), *Desulfococcus* (3.82), *Spirochaeta* (1.64), and ANME-2a/b (2.51) in 16S rRNA gene libraries. There was also a slight enrichment of ANME-2c (1.28). The DSS_658 enrichment had high relative sequence abundance for SEEP-SRB1 (1.74), SEEP-SRB2 (2.78), ANME-2c (1.54), and ANME-2a/b (2.24) with iTag, but these same taxa did not have high relative sequence abundance in the gene library. *Spirochaeta* and SEEP-SRB1 had high relative sequence abundance in both iTag and gene libraries for MSMX-Eel_932 enrichments. Relative sequence abundances for all non-core methane seep taxa in iTag samples are included in Table 3, and where Magneto-FISH enrichments of these additional taxa support network co-occurrences they are discussed in network results.

**Table 3:** iTag relative abundance of remaining ‘non-core’ methane seep microbiome OTUs. Relative sequence abundances were computed for the top 135 OTUs in the iTag dataset that were not included in the core methane seep microbiome. An average and standard deviation for relative sequence abundance among replicates was calculated for each sample set. A ratio of the average relative sequence abundance of Magneto-FISH enrichments compared to the fixed bulk sediment value is reported (Rel. fixed). Ratios over 1.5 are underlined. 16S rRNA gene bacteria and archaea clone libraries for two Magneto-FISH enrichments and fixed bulk sediment are also included for comparison to iTag enrichment.

	Seep1a_1441			DSS_658			Delta_495a			MSMX-Eel_932			Fixed bulk	
Taxon	Avg.	Stdev.	Rel. fixed	Avg.	Stdev.	Rel. fixed	Avg.	Stdev.	Rel. fixed	Avg.	Stdev.	Rel. fixed	Avg.	Stdev.
Desulfarculaceae-uncl	0.02	0.03	2.53	0.02	0.03	2.39	0.01	0.01	1.01	0.05	0.05	7.18	0.01	0.01
*Spirochaetae*-uncl			0.21				0.04	0.02	3.70			0.06	0.01	0.01
Desulfuromusa	0.05	0.05	4.17			0.06					0.01	0.39	0.01	
*Pelobacter*	0.01	0.01	2.48	0.01	0.01	1.95			0.10		0.01	0.81	0.01	
*Actinobacteria*-OM1	0.01	0.01	0.88	0.03	0.01	2.64	0.03	0.04	2.60	0.01	0.01	0.97	0.01	
Alpha-Ancalomicrobium	0.01	0.01	2.29				0.01	0.01	2.50					
*Bacteroidetes*-Actibacter	0.01	0.02	1.38		0.01	0.45	0.01	0.01	0.69			0.06	0.01	
Bacteroidetes-BD-2	0.03	0.01	1.49	0.01	0.02	0.58	0.02	0.01	0.94	0.03	0.02	1.29	0.02	
Bacteroidetes-Lutibacter													0.02	
Bacteroidetes-Marinilabiaceae			3.05		0.01	3.11								
Bacteroidetes-SB-1													0.01	
Bacteroidetes-SB-5	0.01	0.01	0.89	0.01	0.01	0.96						0.70	0.01	
Bacteroidetes-VC2.1_Bac22	0.01	0.01	0.22	0.02	0.01	0.64	0.01	0.02	0.37			0.04	0.03	
Bacteroidetes-WCHB1-69			0.29		0.01	0.30						0.11	0.01	0.01
*Chlorobi*-PHOS-HE36							0.03	0.04						
*Chloroflexi*-Anaerolineaceae	0.02	0.02	0.73	0.01	0.01	0.43	0.01	0.01	0.23	0.02	0.02	0.69	0.03	0.01
Chloroflexi-Bellilinea	0.02	0.03	4.18							0.01	0.01	2.43		
Deferribacteres-*Caldithrix*	0.01	0.01	0.31	0.01	0.01	0.19				0.01	0.01	0.46	0.03	
Deferribacteres-SAR406	0.01	0.01	3.13			0.06	0.03	0.04	8.82			0.18		
Fibrobacteres-uncl		0.01	1.50				0.01		4.82		0.01	1.16		
*Firmicutes*-Fusibacter													0.01	
Firmicutes-Negativicoccus														
Firmicutes-other		0.01	0.59	0.01	0.01	1.15							0.01	
Gam-endosymbionts										0.01	0.01	3.28		
Gamma-other						0.40						0.34		
KB1														
MBGB			0.13	0.01	0.01	1.11	0.01	0.01	1.28			0.66	0.01	
MBGD				0.01	0.01	4.48	0.01	0.01	4.89					
Milano-WF1B-44							0.01	0.02	1.87			0.02	0.01	
OD1	0.02		0.88	0.03	0.03	1.20	0.01	0.01	0.43	0.03	0.01	1.16	0.02	
Plactomycetes-OM190														
*Planctomycetes*-Phycisphaerae	0.01	0.01	0.64							0.02	0.02	2.24	0.01	
Planctomycetes-Pla4														
Planctomycetes-SHA-43											0.01	1.39		
*Sulfurimonas*			0.87							0.01	0.01	1.61		
*Sulfurovum*	0.17	0.16	1.59	0.26	0.11	2.43	0.27	0.18	2.49	0.06	0.03	0.52	0.11	0.01
TA06		0.01	1.12											
Thaumarc-uncl						0.12							0.01	
*Thiohalobacter*				0.01	0.01									
Thiotrichaceae-uncl														
WS3	0.01	0.01	0.47	0.04	0.04	2.21	0.02	0.03	1.20	0.03	0.01	1.74	0.02	

### Statistical evaluation of Magneto-FISH enrichment

To statistically compare enrichment microbial communities, we used a suite of statistical tests including: non-parametric *T*-tests (White, Nagarajan & Pop, 2009), LEfSe (Segata et al., 2011), and UniFrac (Lozupone & Knight, 2005). Using the *T*-test comparison, ten OTUs were significantly (*p* < 0.001) different between the bulk sediment and Magneto-FISH samples (when only including OTUs with sequences present in both groups). The taxonomic assignments for these ten OTUs were: WCHB1-69, *Desulfobulbus*, *Thaumarcheota*, ANME-1a, *Bacteroidetes* (VC2.1), ANME-2c, *Caldithrix*, SEEP-SRB1, Candidate Division TA06, and *Gammaproteobacteria* (CS-B046). LEfSe was then used to determine which OTUs were significantly different between Magneto-FISH enrichments and bulk sediment. We found three OTUs were significantly (*p*-value < 0.05) higher in relative sequence abundance in Magneto-FISH samples over bulk sediment with the taxonomies: SEEP-SRB1, *Desulfobulbus*, and *Planctomycetes* (SHA-43).

Weighted UniFrac analysis was used to compare the community composition between Magneto-FISH iTag enrichments. The UniFrac metric represents the fraction of the branch length that is unique to each sample, or unshared between samples, such that a higher ratio means less similar samples. The *Deltaproteobacteria* probe enrichment communities were more similar to each other than any of the *Deltaproteobacteria* probes compared with the MSMX-Eel_932 probe (Table 4). The most distinct communities were MSMX-Eel_932 enrichment and Delta_495a enrichment, with the highest proportion of unshared branch length (0.97; *p*-value < 0.001). MSMX-Eel_932 enrichment and DSS_658 enrichment had less unshared branch length at 0.88 (<0.001), suggesting MSMX-Eel_932 and DSS_658 probes enrich for a more similar community than MSMX-Eel_932 and Delta_495a probes. Comparison of the MSMX-Eel_932 enrichment and SEEP-1a_ 1441 enrichment communities was not significant at the <0.001 cutoff. Within the *Deltaproteobacteria* probes, SEEP-1a_1441 enrichment and DSS_658 enrichment had the lowest proportion of unshared community (0.77, <0.001); the most similar community structures were recovered with these two probes. The next lowest proportion of unshared community is between DSS_658 enrichment and Delta_495a enrichment (0.81). SEEP-1a_1441 enrichment and Delta_495a enrichment are least similar, at 0.85. All of these values are highly significant (<0.001). This is consistent with the expectation that the overlap between the target microbial population of the SEEP-1a_1441 probe would be most similar to the target microbial population of the DSS_658 probe, while the Delta_495a enrichment would recover more total *Deltaproteobacteria* diversity.

**Table 4:** CARD-FISH aggregate counts. Aggregate counts from triple CARD-FISH hybridizations with probes targeting ANME-1 (ANME- 1_350), all ANME (Eel_932), DSS type *Deltaproteobacteria* (DSS_658), *Epsilonproteobacteria* (Epsi_404), SEEP-SRB1a (SEEP-1a_1441) and *Cytophaga*, *Bacteroidetes*, *Flavobacterium*, and *Sphingobacterium* (CF_319A/B) associations.

	ANME-1_350	Eel_932	DSS_658	Epsi_404	Gam_42a	Seep-1a_1441	CF_319A/B
Total	39	70	91	5	12	29	8
With ANME-1			36	2	6	21	0
With ANME-2			63	1	9	21	4
Percent of all	39%	70%	91%	10%	24%	58%	16%
Percent ANME-1			36%	4%	12%	42%	0%
Percent ANME-2			63%	2%	18%	42%	8%

### Assessing community structure with co-occurrence network analysis

After determination of statistically significant differences between iTag Magneto-FISH and bulk sediment samples, we computed co-occurrence networks to observe which of the 135 most abundant OTUs were correlated in the methane seep microbial community. By combining the results from 100 separate microbial association calculations, we were able to assign confidence to each microbial association and determine the most robust associations. Significant associations are reported in Table S5 and depicted as a network in Fig. 1.

Focusing first on the common ANME syntrophic *Deltaproteobacteria* partner, SEEP-SRB1, this taxon had the most associations in the network including nine positive associations and one negative association (Fig. 1). There are two separate sets of SEEP-SRB1 & *Planctomycetes* (AKAU3564 sediment group) positive associations that are both well supported. SEEP-SRB1 is also associated with three other heterotrophic taxa (Candidate Phylum Atribacteria, *Spirochaeta*, and *Bacteroidetes* (VC2.1_ Bac22)) and one sulfur-oxidizing taxa (*Sulfurovum*). SEEP-SRB1 was also associated with Candidate Division Hyd24-12, which has a currently unknown ecophysiology, but could be a heterotroph if the topology of heterotrophic taxa being in the center of the network holds true. Hyd24-12 and *Atribacteria* are also both associated with the second most associated taxa, Candidate Division OD1, but there was no direct association between SEEP-SRB1 and OD1. SEEP-SRB2 has two of the same associations as SEEP-SRB1 (VC2.1_Bac22 and *Atribacteria*), but is the only *Deltaproteobacteria* associated with MBG-B, *Anaerolineaceae*, and *Desulfoluna* (another *Deltaproteobacteria*). SEEP-SRB4 is associated with *Desulfobulbus*, and the only *Deltaproteobacteria* associated with and ANME (2a/b), WS3, and *Actibacter*. WS3 had high relative sequence abundance in both DSS_658 and MSMX-Eel_932 enrichments (Table 3). *Desulfobulbus* is associated with *Desulfococcus*, the only *Deltaproteobacteria* associated with BD2-2, and SAR406. SAR406 had high relative sequence abundance in Seep1a_1441 and Delta_495a enrichments (Table 3). The heterotroph *Spirochaeta* is also included in the core methane seep microbiome and was associated with *Clostridia* and WS3, in addition to Hyd24-12 and SEEP-SRB1.

In examination of additional OTUs associated with sulfur metabolisms, we found *Sulfurovum* and *Sulfurimonas* (*Epsilonproteobacteria*) were not associated with each other, but are both associated with *Deltaproteobacteria*. *Sulfurimonas* is associated with *Desulfocapsa* and *Sulfurovum* is associated with SEEP-SRB1 and *Desulfobulbus*. *Sulfurovum* had high relative sequence abundance in MSMX-Eel_932 enrichments and *Sulfurimonas* had high relative sequence abundance in Seep-1a_1441, DSS_658, and Delta_495a enrichments (Table 3). The *Gammaproteobacteria*, *Thiohalobacter*, is only associated with *Anaerolineaceae* and was not elevated in any of the Magneto-FISH enrichments.

Heterotrophs are the most dominant metabolic guild in the network, and similar to sulfate-reducers, have some of the most connected taxa. The heterotroph OD1 has seven positive correlations, in addition to *Atribacteria* and Hyd24-12 listed above: *Bacteroidetes* (BD2-2), *Actinobacteria* (OM1), *Pelobacter*, ANME-1b, *Chloroflexi* (*Anaerolineaceae)*, and *Desulfocapsa. Anaerolineaceae* and *Bacteroidetes* (BD2-2) both had seven associations, but with different connectivity. BD2-2 was interconnected with other heterotrophs, sulfate-reducers, and archaeal methanotrophs in the main portion of the network, whereas *Anaerolineaceae* was connected to three taxa that share no other connections (two heterotrophs and one *Gammaproteobacteria* sulfur oxidizer). The one other ANME taxa in the network, ANME-1b, is only positively associated with heterotrophs and no known sulfate reducing groups.

### Assessing ANME-bacterial partnerships by CARD-FISH

To assess ANME and DSS relative cell abundance, 100 aggregate clusters from the same sediment incubation (see ‘Materials & Methods’) were analyzed with CARD-FISH and the DSS_658/ANME1-350/MSMX-Eel_932 probe combination. Epsi_404, Gam_42a, SEEP-1a_1441, and CF_319A/B probes were also used with the archaeal probe combination to examine non-DSS bacterial diversity recovered in the network analysis ANME associations. All probes, target populations, and references are listed in Table 1.

**Figure 2:**
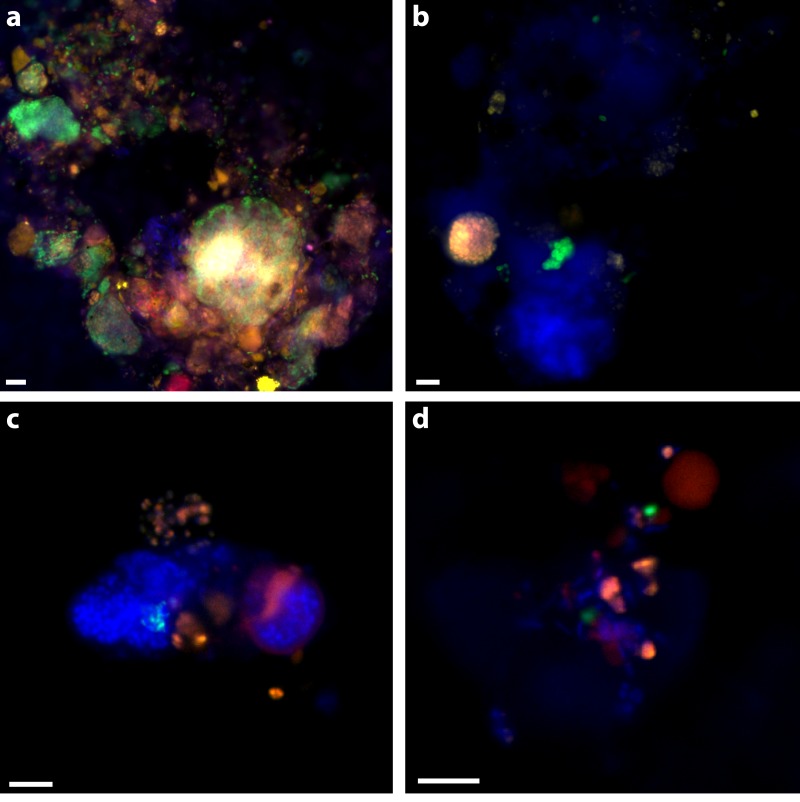
Examples of triple CARD-FISH hybridized aggregates. Triple CARD-FISH hybridization using bacteria and archaea probes targeting DSS_658 (A), Gam42a (B), CF319A/B (C), and Epsi404 (D) in green FITC, with ANME1-350 in red and MSMX-Eel_932 in yellow for all. Scale bar 5 µm for all. DAPI stain in blue.

30% of aggregates contained an ANME-2 signal (see ‘Materials & Methods’; Table 5) and 39% of aggregates had an ANME-1 signal. ANME-1 and ANME-2 identified cells were also consistent with expected morphologies. Multiple clusters of mixed-type ANME/DSS, DSS-only, ANME-only, DSS/non-ANME, and non-DSS/non-ANME aggregates were observed with the ANME-1_350, MSMX-Eel_932, and DSS_658 probe combination (Fig. 2A). There were no clear examples of aggregates with ANME/non-DSS hybridized cells, though we found many instances where both ANME and non-DSS cells were as part of a larger aggregate cluster with other cell types. ANME-1 cells often occurred in the matrix surrounding tightly clustered ANME-2 aggregates. The SEEP-1a_1441 probe, targeting a subgroup of DSS, was observed to hybridize with aggregate clusters that contained ANME-1 and ANME-2 cells, but usually with SEEP-SRB1/ANME-2 in tight association and ANME-1 in more peripheral association. Five of the SEEP-SRB1/ANME-2 aggregate clusters did not have ANME-1 cells (10%) and three of the SEEP-SRB1/ANME-1 aggregate clusters did not have ANME-2 cells.

**Table 5:** UniFrac analysis of Magneto-FISH samples. Community comparison of iTag Magneto-FISH samples using weighted UniFrac analysis.

	Seep1a1441	DSS658	Delta495a	Eel932
Seep1a1441	–	0.77^∗^	0.85^∗^	0.91 +
DSS658	–	–	0.81^∗^	0.88^∗^
Delta495a	–	–	–	0.97^∗^

**Notes.**

Significance of relationship between communities is reported with *p*-values: ^∗^, <0.001; ˆ, 0.002; +, 0.030.

Ten percent of aggregates (*n* = 50 counted) hybridized with the Epsi_404 probe, broadly targeting members of the *Epsilonproteobacteria*. These *Epsilonproteobacteria* were mostly found in association with other bacteria and occasionally, loosely associated with some ANME. Epsi_404 hybridized cells were generally ovoid and scattered throughout an EPS matrix of cells, as depicted in Fig. 2D. There was no apparent preference for *Epsilonproteobacteria* association with ANME-1 or ANME-2 aggregate clusters (Table 4). A higher percentage of aggregates had *Gammaproteobacteria* cells (24% of 50) than *Epsilonproteobacteria* cells, and there was a slightly higher co-occurrence with ANME-2 (18%) than ANME-1 (12%) hybridized cells. The dominant *Gammaproteobacteria* morphology observed was a cluster or chain of large (∼1 µm) ovoid cells. Gam_42a hybridizing cell clusters and chains were found both separately and associated with other bacteria, as in Fig. 2B, where they are predominately an unidentified cluster stained by DAPI with a sub-aggregate of ANME-2 cells. CF319A and CF319B were used to target *Cytophaga*, *Bacteroidetes*, *Flavobacterium*, and *Sphingobacterium*. Eight percent (*n* = 50 counted) of aggregates contained cells positively hybridizing with the CFB probe, generally observed as clustered filaments or rods (Fig. 2C). Half of these aggregates also had ANME-2 hybridized cells. No CFB cells were observed to co-associate with ANME-1.

## Discussion

### Evaluation of Magneto-FISH with iTag

Challenges accompanying downstream analysis of Magneto-FISH enrichments are primarily associated with low DNA yield and poor DNA quality from aldehyde fixation (for further discussion of fixation effects see Trembath-Reichert, Green-Saxena & Orphan, 2013). Low template concentration exacerbates amplification of contaminating sequences since target and non-target templates can approach parity in a PCR reaction. Low template concentration has also been shown to create random variation in amplification products in dilution experiments (Chandler, Fredrickson & Brockman, 1997), which could explain the high variation seen in Magneto-FISH enrichment relative sequence abundances compared to bulk sediment samples. Despite these challenges, the DNA recovered from Magneto-FISH enrichments has been shown to increase the sequence abundance of target organisms relative to the bulk sediment by 16S rRNA gene sequencing and metagenomics on various Next Generation sequencing platforms (Pernthaler et al., 2008; Trembath-Reichert, Green-Saxena & Orphan, 2013). In this study, conventional cloning and sequencing of full-length bacterial and archaeal 16S rRNA genes had fewer contamination issues as compared to iTag sequencing with universal primers. Our Magneto-FISH experiments were designed to mitigate as many sampling and iTag sequencing biases between samples as possible, by concurrently extracting, amplifying, and sequencing all Magneto-FISH samples in parallel, including biological and technical replicates. The relative ratio of contaminant reads to environmental OTU’s were higher in Magneto-FISH enrichments than in bulk sediment samples, but bulk sediment could be used to separate indigenous community members from putative contaminants in the Magneto-FISH samples (see ‘Materials & Methods’). This provided a conservative Magneto-FISH dataset for statistical analyses and demonstrated the importance of parallel processing sequencing of bulk and separated samples.

In addition to issues with contaminating sequences, we also observed bias against some core methane seep microbiome taxa, where these taxa were consistently underrepresented by iTag when compared to gene libraries and CARD-FISH. ANME-2 was the most underrepresented taxon in iTag sequencing of the bulk sediment and mock communities, with much greater relative sequence and relative cell abundance in gene library sequencing and CARD-FISH analysis, respectively. It is most likely that iTag sequencing bias with the EMP primer set is the reason ANME-2c was not enriched in the Magneto-FISH samples and absent from microbial community network analysis. Members of the ANME-2a/b were also, to a lesser extent, underrepresented with iTag. In addition to our gene libraries and CARD-FISH analysis, independent assays using FISH with mono labeled oligonucleotide probes from this sediment incubation further confirmed the abundance of ANME-2 aggregates; 25% of aggregates were ANME-2c and 17% of aggregates were ANME-2b, with about half of ANME-2 aggregates associating with a bacterial partner other than SEEP-SRB1 (Supplement McGlynn et al., 2015). We conclude that while expected ANME-2 associations were not recovered, they can be explained by EMP iTag bias and therefore do not reduce the validity of other non-ANME-2 associations recovered in the co-occurrence analysis (see Tables S2 and S3 captions for further discussion of ANME-2c bias). Although ANME-1a was not underrepresented in the iTag data, it still does not appear in the co-occurrence network. In other co-occurrence network studies dominant OTUs were not associated with the majority of the microbial community, which was thought to be due to a high degree of functional redundancy (Mu & Moreau, 2015). Possible functional redundancy with other archaeal groups, or simply non-specific, loose spatial association with many taxa, as suggested by CARD-FISH analysis, could explain why ANME-1a was not recovered in our network analysis.

Despite this unanticipated methodological bias, iTag sequencing is a valid and valuable tool when combined with Magneto-FISH enrichment techniques for microbial association hypothesis development and testing. For example, we saw more bacterial OTUs, especially among *Deltaproteobacteria*, in the iTag samples compared with conventional gene libraries and the core methane seep taxon Hyd24-12 was not even observed among gene library sequences.

### Magneto-FISH enrichment

This study provides a novel combination of nested Magneto-FISH enrichments and microbial community network analysis methods to develop hypotheses regarding specific lineage associations and, by inference, discusses the potential for additional metabolic interactions relating to sulfur cycling in methane seep sediments. Notwithstanding the low recovery of ANME-2 OTUs, there was statistical support for Magneto-FISH enrichments increasing the relative iTag sequence abundance of target organisms. Statistical analyses demonstrated SEEP-SRB1 and *Desulfobulbus* OTUs were significantly different in Magneto-FISH samples (*t*-tests), and these OTUs were significantly more enriched in Magneto-FISH samples using linear discriminant analysis (LDA) effect size (LEfSe). Additionally, weighted UniFrac analysis showed the highest percentage of shared phylogeny was between the clade-specific SEEP-1a_1441 probe and the family-specific *Desulfobacteraceae* DSS_658 probe enrichments. Therefore these Magneto-FISH samples contain microbial community overlap consistent with probe target specificity, even when some dominant community members are not represented at expected relative sequence abundance in the iTag analysis (ANME-2).

Magneto-FISH enrichment relative sequence abundance followed expected trends for *Deltaproteobacteria* (Table 2). SEEP-SRB1 had the highest relative sequence abundance in Seep-1a_1441 and MSMX-Eel_932 enrichments, which should target this group. *Desulfobulbus* had the highest relative sequence abundance in the Delta_495a enrichment, which was the only Magneto-FISH probe that should hybridize to this group (though *Desulfobulbus* could also be retrieved via association with other target organisms). OTUs affiliated with *Desulfoluna* (within the *Desulfobacteraceae*) had the highest relative sequence abundance of all *Deltaproteobacteria* in the DSS_658 enrichment and are also targeted by the DSS_658 probe. *Desulfoluna* were not specifically targeted by MSMX-Eel_932 or Seep-1a_1441 probes, but had high relative sequence abundane in these samples and may have a potential association with ANME/DSS consortia. Also, *Atribacteria* (JS1) was recovered in all iTag sequencing of Magneto-FISH enrichments, suggesting they may associate with either DSS/ANME or DSB/ANME consortia. Members of the Hyd24-12 were only recovered in Seep1a_1441 and MSMX-Eel_932 enrichments and may preferentially associate with SEEP-SRB1a/ANME consortia.

Evaluating our iTag relative sequence abundance data with co-occurrence analysis, we developed hypotheses that were not subject to the variation between Magneto-FISH enrichment replicates; associated taxa should always co-vary, even when they are less abundant than expected. Within the core methane seep taxa, high relative sequence abundances of *Atribacteria* and Hyd24-12 with SEEP-SRB1 targeting Magneto-FISH enrichments were upheld by the network. Hyd24-12 is highly associated with SEEP-SRB1, whereas *Atribacteria* is highly associated with both SEEP-SRB1 (DSS) and SEEP-SRB2 (DSB). While *Atribacteria* have not been cultured, metagenomic sequencing suggests they are likely heterotrophic anaerobes involved in fermentation (Nobu et al., 2015). Hyd24-12 was first cloned from Hydrate Ridge (Knittel et al., 2003) and has been cited as a core methane seep microbial taxon (Ruff et al., 2015), but nothing is known about its physiology. The Hyd24-12/SEEP-SRB1 association was also one of the four unique associations that were recovered in all the network computations (*n* = 100). These results may aid in determining a role for these enigmatic candidate phyla of the methane seep microbiome.

*Methanomicrobia* and *Deltaproteobacteria* only had one co-occurrence in our network. The one statistically supported network ANME/SRB association was between ANME-2a/b and SEEP-SRB4. SEEP-SRB4, belonging to the *Desulfobulbaceae* (Knittel et al., 2003), and ANME-2a/b both had high relative sequence abundance in the ANME-targeting MSMX-Eel_932 enrichment bacterial 16S rRNA gene library. There have been FISH-confirmed physical associations between ANME-2/ANME-3 and *Desulfobulbaceae* (Green-Saxena et al., 2014; Löesekann et al., 2007; Pernthaler et al., 2008) in AOM systems. SEEP-SRB4 was also strongly associated with the candidate phyla WS3 in the network, and WS3 was enriched in both DSS_658 and MSMX-Eel_932 enrichments. Both SEEP-SRB4 associations with ANME-2a/b and WS3 warrant future study.

While expected ANME-2/*Deltaproteobacteria* associations were not recovered (see *Evaluation of Magneto-FISH with iTag*), network analysis did recover many *Deltaprotobacteria* co-occurring with bacterial groups. Almost half of all positive associations contained a *Deltaproteobacteria* OTU (30/61), suggesting a dominant role for the sulfur cycle metabolisms. Of those, 21 associations were with a non-*Proteobacteria* OTU including a number of candidate organisms as described above. The association between SEEP-SRB1 and ‘AKAU3564,’ a *Planctomycetes*-affiliated heterotrophic sediment group, was observed twice with two separate OTU associations in this clade that were both strongly supported (occurring 100/100 and 93/100 times, respectively, that the network analysis was run, Table S5). This Planctomycete group was first described in methane hydrate bearing deep marine sediments of the Peru Margin (Inagaki et al., 2006). *Planctomycetes*-associated sequences were previously recovered in association with ANME-2c Magneto-FISH samples from the Eel River Basin, where the preferred partner was observed to be the SEEP-SRB1 group (Pernthaler et al., 2008). It follows that SEEP-SRB1 may also co-occur with *Planctomycetes*, if these organisms are affiliated (either directly or indirectly) with ANME-2 consortia. By similar logic, although it did not have high relative sequence abundance in the Seep1a_1441 enrichment, this could explain the high relative sequence abundance of this group in the MSMX-Eel_932 enrichment (Table 3). *Planctomycetes* targeted CARD-FISH hybridization using the general *Planctomycetes* probe Pla_886 was attempted; however, many cells with a morphology similar to ANME-1 were hybridized and the results were deemed inconclusive. This ambiguity could be due to the probe’s single base pair mismatch to 97% of ANME-1a, 94% of ANME-1b, and 25% of ANME-2b, even if this mismatch was centrally located (SILVA TestProbe online tool, Greuter et al., 2016). *Spirochaeta* was also associated with SEEP-SRB1, in addition to Hyd24-12 and WS3, and had high relative sequence abundance in both the DSS_658 and MSMX-Eel_932 enrichments (Table 2). In addition to being core methane seep microbial taxa, some members of the *Spirochaetes* have sulfide-oxidizing capabilities in mats with sulfidogenic bacteria (Dubinina, Grabovich & Chernyshova, 2004) and its possible that these organisms may be utilizing sulfide produced in seep systems as well.

*Epsilonproteobacteria* and *Deltaproteobacteria* were the most common intra-*Proteobacteria* association in the network and have been shown to co-occur in many sulfidic habitats (Campbell et al., 2006; Omoregie et al., 2008), where *Epsilonproteobacteria* oxidize sulfur and *Deltaproteobacteria* disproportionate or reduce sulfur species (Pjevac et al., 2014). In the network, *Sulfurovum* was associated with both SEEP-SRB1 and *Desulfobulbus*, and this was also seen in the relative sequence abundance data where *Sulfurovum* had high relative sequence abundance in all of the *Deltaproteobacteria* Magneto-FISH enrichments. *Epsilonproteobacteria* have been shown to oxidize sulfide to S° or HS^−^ to sulfate in microbial mats (Pjevac et al., 2014), allowing some sulfur substrate differentiation between these *Epsilonproteobacteria* groups in this system. *Sulfurimonas* was not strongly associated with any *Deltaproteobacteria* in the network analysis and only had high relative sequence abundance in the MSMX-Eel_932 enrichment (16S rRNA gene iTag, 16S rRNA gene bacterial, and soxB gene libraries; see Fig. S2 for further discussion of metabolic genes). CARD-FISH analysis using probe Epsi_404 confirmed the presence of *Epsilonproteobacteria* cells within some ANME and other non-hybridized cell-containing loose aggregates, but did not appear to be in the tight physical association characteristic of ANME/SRB consortia. While cultured representatives of these *Epsilonproteobacteria* have optimum growth with some oxygen present (Inagaki et al., 2003; Inagaki et al., 2004), it is possible that these uncultured methane seep *Epsilonproteobacteria* may be able to use other oxidants such as nitrate or intermediate sulfur species while in anaerobic incubation conditions.

In comparison to *Delta*- and *Epsilonproteobacteria*, there was only one *Gammaproteobacteria* OTU in the network (*Thiohalobacter*, with one *Anaerolineaceae* association). Cultured representatives of *Thiohalobacter* have diverse sulfur capabilities, including thiocyanate metabolism, but are not known to form associations with other sulfur cycling organisms (Sorokin et al., 2010). This differentiation between *Gamma*- and *Epsilon*-/*Deltaproteobacteria* has been seen in other systems such as sulfidic cave biofilms (Macalady et al., 2008) or in microbial mats on marine sediments (Pjevac et al., 2014). Gam_42a hybridizing cells (*Gammaproteobacteria*) were observed to form aggregates with non-ANME and non-*Desulfobulbaceae* (DSS) cells in our CARD-FISH analysis, but the identity of these organisms was not determined. While not recovered in the network, the majority of the *Gammaproteobacteria* OTUs observed by iTag from the both the bulk sediment and MSMX-Eel_932 Magneto-FISH 16S rRNA gene (Table 1) and aprA gene libraries (see Fig. S2 for further discussion of metabolic genes) were from the SILVA taxonomy endosymbiont clade. This endosymbiont clade houses organisms with a carbon-fixation/sulfur-oxidation metabolism (Duperron et al., 2012; Goffredi, 2010) and is predicted to be an important member of the sulfur and carbon cycles in marine sediments outside of an endosymbiotic lifestyle (Lenk et al., 2011).

There were also three unique, positive *Deltaproteobacteria-Deltaproteobacteria* associations observed in the network (*Desulfobulbus*/*Desulfococcus*, *Desulfobulbus*/SEEP-SRB4, *Desulfoluna*/SEEP-SRB2). These multiple intra-*Deltaproteobacteria* associations suggests there may be further nuances to be explored in the *Deltaproteobacteria* community structure, perhaps akin to the nitrate based partitioning observed between DSB and DSS in seep sediments (Green-Saxena et al., 2014). *Desulfobulbus* was also associated with SAR406, and SAR406 had high relative sequence abundance in the Delta495a enrichments. SAR406 (Marine Group A) fosmids contained polysulfide reductase genes that may be used for dissimilatory polysulfide reduction (Wright et al., 2014) *Desulfobulbus* can also use polysulfide, in addition to a range of other sulfur sources (Fuseler & Cypionka, 1995), potentially linking these two taxa.

## Conclusions

Our findings support the utilization of paired Magneto-FISH and iTag sequencing in developing and testing hypotheses to interrogate complex interactions in microbial communities. Contaminants and amplification bias can be identified and mitigated with diversity assessment by multiple means (i.e., multiple iTag primer sets, FISH surveys, or non-16S rRNA gene surveys) and parallel processing of control samples (bulk sediment and no-template) along with Magneto-FISH enrichments. Since it may not always be known *a priori* which taxa are in an environmental sample, sequencing of a defined mock community may not be an option for assessing bias. However in our case, prior knowledge of major seep taxa enabled assessment of amplification bias by iTag. It should also be noted that the degree of bias was more pronounced in the environmental samples than our mock samples, therefore mock community samples may not fully capture the degree of bias, but can be useful in identifying which taxa may be the most biased. We found the bulk sediment 16S rRNA gene libraries to be the most useful for determining which of the most abundant taxa were affected by amplification bias. Future studies may benefit more from bulk sediment analysis by a range of iTag primer sets or gene libraries to assess potential sequencing biases in a new microbial community.

Multiple statistical methods supported differences between Magneto-FISH enrichments and the bulk sediment. We also found variation between SparCC network computations. Therefore, we added confidence to network associations by reporting the number of times an association was recovered out of 100 co-occurrence iterations along with correlation and *p*-value.

Our resultant microbial community network had many statistically significant methane seep taxa correlations beyond the common ANME/SRB association. The downplay of anaerobic methanotrophs in our iTag sequencing may have had the beneficial effect of bringing fermenters to the forefront, highlighting their complex role in methane seep microbial communities. Within the core methane seep microbiome taxa, there were strong associations between *Atribacteria* and Hyd24-12 and *Deltaproteobacteria*, but no direct association between *Atribacteria* and Hyd24-12. This may indicate a different niche for these two currently uncultured groups in methane seep systems. *Sulfurovum* and *Sulfurimonas* were differentiated as either *Deltaproteobacteria*-associated or archaea-associated, respectively. There were statistically significant associations between *Deltaproteobacteria* and non-*Proteobacteria*, such as the *Planctomycetes* sediment group ‘AKAU3564,’ and groups that contained neither SRB nor ANME but had high statistical significance, such as MBG-B and OM1. Future development and application of more specific FISH probes will assist in further hypotheses development and testing of these associations in Hydrate Ridge methane seeps.

Some groups, such as *Gammaproteobacteria*, appeared to have associations with other microbes based on broad FISH surveys and Magneto-FISH relative sequence abundance data, but were not recovered in the network analysis. Determination of the specific *Gammaproteobacteria* involved in associations via FISH probe development or other means (Hatzenpichler et al., in review) will also aid in refining why associations might be missed in the microbial network analysis based on DNA taxa co-occurrence. In summary, a continual feedback loop between microbial identification and isolation techniques and gene based statistical analyses is required to tease apart interactions within complex microbial systems. The combination of Magneto-FISH and high throughput, parallel iTag sequencing provides an effective bridge between these two modes.

## Supplemental Information

10.7717/peerj.1913/supp-1 Table S1 Extracted DNA Extracted DNA concentration per sample measured by fluorometer.

10.7717/peerj.1913/supp-2 Table S2 Mock sediment community sequence recovery Expected and recovered sequence abundances among the mock communities show differential taxonomic biases. *Fold Change* is calculated by dividing the experimentally recovered relative abundance by the expected relative abundance. Four mock communities were designed with a selection of common methane seep bacterial and archaeal taxa at different relative abundance ratios. Mock community analysis revealed that relative abundances of *Helicobacteraceae* (*Sulfurovum*), *Desulfobacteraceae* (Seep-SRB1) and *Desulfobulbaceae* (*Desulfobulbus*) had little amplification bias as compared to other mock community taxa (fold change ranges 0.93–1.42, where 1.00 means expected relative abundance was returned). ANME-1b plasmids were also overall well represented (fold change 0.64 to 1.42) across the range of expected relative abundances (1% to 20%). In contrast, ANME-2a/b and ANME-2c plasmids were always under amplified in all of the mock communities (fold change 0.32 to 0.81). These results do not appear to correlate to primer hits in the SILVA SSU r123 database, where 89.5% of ANME-2c sequences were hit by 515f and 87.1% by 806r, but 94.3% of ANME-2a/b were hit by 515f and 806r. ANME-2a/b was a better match to the EMP primers, but both taxa were under amplified in mock community analysis. Amplification bias was not always uniform, where some templates saw varied amplification response depending on initial relative abundance in the mock community. The ANME-1a plasmid was over-amplified (3.35–2.44 fold change; Table S2) when the plasmid was at 5% relative abundance and lower (Mock Communities 1–3). However, Mock Community 4 with the highest relative abundance (20%) of ANME-1a plasmids, saw templates amplified to the expected relative abundance (0.97 fold change). *Thaumarchaeota*: miscellaneous *Crenarchaeota* Group followed a similar pattern to ANME-1a: where it was 1% expected relative abundance, the fold change is ∼5, and where it was 10% expected relative abundance, the fold change was less pronounced (∼1.5). MBG-D sequences were slightly over amplified when at 1% expected relative abundance, and slightly under amplified when at 42% relative abundance. Bias was consistent across mock community samples when the relative percentage of that group (e.g., *Thermoplasmatales*, 40%) was the same in both samples. This suggests that analysis based on relative abundance between samples can be applied as a means of comparison, as long as the environmental OTUs of interest are above the detection threshold. A study of EMP primers with a pelagic marine community also reported discrepancies between mock community bias and independently assessed environmental sample bias for a dominant community members (Parada et al., 2015). Parada et al. similarly conclude that over-amplification of certain community members, in their case *Gammaproteobacteria*, was the cause of lower than expected recovery, rather than lack of SAR11 and SAR116. Our ANME-2c results, therefore, serve as yet another example of how key community members can be under-represented when exploring unknown microbial systems. The severity of this issue for future studies is dependent on the research question, interpretation approach, and the phylogenic bias imparted on community members of interest. The phenomenon of less pronounced bias when templates are at higher starting relative abundances could be explained by the reannealing inhibition affect of high copy number templates in mock samples (Suzuki & Giovannoni, 1996). Due to low template of Magneto-FISH samples, PCRs were done for a total of 35 cycles. Since bias is positively correlated with number of cycles (Suzuki & Giovannoni, 1996), lowering PCR amplification cycles could improve bias issues. The lack of statistically significant ANME-2c correlations is expected since this group was recovered in so few samples. ANME-1a, however, may suffer from the opposite problem where over-amplification in iTag datasets reduces the ability to determine patterns with other OTUs. As an analogy, if the ANME-2c population is an image with only a few pixels and the image of the ANME-1a population is an image with oversaturated pixels, then neither has a workable dynamic range for correlation analysis. The log transform operation performed on the data before correlation analysis can reduce the bias between high and low abundance OTUs to some degree, but may not be sufficient in all cases, such as with these two OTUs. Several approaches can ameliorate some of the issues within iTag sequencing datasets: (1) Optimization of PCR conditions and use of high-fidelity DNA polymerase for amplification in conjunction with the (2) creation and sequencing of a mock community, if there is *a priori* knowledge of the community composition; (3) Data transformation(s) before statistical analysis (i.e., square root, fourth root, or log transformations) and (4) examining the behavior of single OTUs across multiple samples/treatments may be more robust than direct comparison of OTUs within a single sample; (5) Whole-community comparisons (i.e., UniFrac, ANOVA, ANOSIM), to minimize single-taxon biases by including all taxa.

10.7717/peerj.1913/supp-3 Table S3 Sequences per sample post processing Total sequences per sample after *mothur* processing and 0.1% bulk sediment cutoff and total sequences remaining for the most abundant ANME-2c, SEEP-SRB1, and ANME-1a OTUs. We also performed a BLASTN (Maddenm 2002) search of all contigs from all samples against an in-house database of 155 ANME-2c 16S rRNA sequences of >500 bp. This yielded 1,395 iTag sequences with an *e*-value greater than or equal to 10^−130^, corresponding to 99–100% sequence identity match to sequences from our ANME-2c database. We then tracked this set of BLAST match contigs through each step in the *mothur* pipeline, with a final result of 1,260 sequences remaining in this BLAST set from the original contig file. Thus 92% of our ANME-2c BLAST hit set remained through the *mothur* processing pipeline. This suggests that the lack of ANME-2c sequences in our downstream database was not due to spurious removal during sequence processing.

10.7717/peerj.1913/supp-4 Table S4 Mock community sequencing error rates Mock Community sequencing error rates (0.025–0.095%; Table S4) were of the same magnitude as Kozich et al. (∼0.01%, 2013). Rarefaction of the mock community to 5,000 sequences shows OTU inflation rates of 3 to 4 times expected number of OTUs, after 97% OTU clustering and removal of singletons. The inflation rate is calculated by total number of OTUs recovered divided by original number of template plasmids. Since our environmental mock community only had 12 templates, the number of spurious OTUs is expected to be high (Huse et al., 2010). Experimental sediment samples have 10 to 100 times more templates, so inflation rates are expected to be much lower (10–1%).

10.7717/peerj.1913/supp-5 Table S5 Combined network associations All associations that occurred in at least 50 out of 100 networks for combined Magneto-FISH and bulk sediment samples with their number of occurrences, average correlation, and *p*-values.

10.7717/peerj.1913/supp-6 Figure S1 Flow diagram of methods

10.7717/peerj.1913/supp-7 Figure S2 Comparative phylogenetic trees from dsrA, aprA, and soxB functional genes Phylogenetic trees of SoxB, AprA, and DsrA functional genes from a MSMX-Eel_932 Magneto-FISH enrichment. aLRT SH-like values above 50% displayed for branch support. Similar sequence clusters represented by one sequence are indicated in parentheticals, and sequences from this study are in bold. Clones recovered are also summarized in table form. As another method of assessing Magneto-FISH diversity, we examined functional genes relating to the sulfur cycle. This method can also provide insight into phylogenetic connections between 16S rRNA and sulfur cycling functional genes. Clone libraries were constructed from an MSMX-Eel_932 Magneto-FISH capture and performed with sediment as iTag libraries (see ‘Materials & Methods’). The following genes relating to sulfur cycling pathways were chosen for this analysis: soxB (sulfur oxidation, protein-S-thiocysteine sulfate hydrolase), aprA (sulfur oxidation and reduction, adenylylsulfate reductase *α* subunit), and dsrA (sulfur oxidation and reduction, dissimilatory sulfite reductase). Phylogenetic analysis of soxB clones from the MSMX-Eel_932 Magneto-FISH returned only *Epsilonproteobacteria* sequences from both *Sulfurovum* (2 clones) and *Sulfurimonas* (20 clones) clades (Fig. S1). From a total of 13 aprA clones, 7 were retrieved from the *Desulfobacteraceae* clade (SEEP-SRB1 containing), none from the *Desulfobulbaceae*, 5 from *Gammaproteobacteria* Endosymbiont clade, and 1 from the “Cluster B” GoM clone clade (Meyer & Kuever, 2007). 15 of 16 dsrA clones were from the *Desulfobacteraceae* clade, with one clone from the *Desulfobulbaceae* clade (Müller et al., 2015). Functional gene clone libraries were not only successful in providing another means to assess Magneto-FISH enrichment, but provide an example of how this technique can be utilized to target specific 16S rRNA populations and the metabolic diversity contained. This is particularly useful in cases where 16S rRNA and functional gene phylogenies are not well aligned.

## Additional Information and Declarations

### Data availability

The following information was supplied regarding data availability: GenBank sequences: AprA ( KT280505– KT280517), DsrA ( KT280518– KT280533), McrA ( KT280534– KT280581), Archaeal 16S rRNA ( KT280582– KT280632), Bacterial 16S rRNA ( KT280633– KT280909), SoxB ( KT280910– KT280928) SRA: accession SAMN03879962, BioSample: SAMN03879962, Sample name: PC47 (5133–5137) mixed slurry.
