# Supplementary material for: Characterization of microbial associations with methanotrophic archaea and sulfate-reducing bacteria through statistical comparison of nested Magneto-FISH enrichments
Source: PeerJ. 2016 Apr 18;4:e1913. doi: 10.7717/peerj.1913 (PMC4841229; doi:10.7717/peerj.1913)
Supplement: Table S1 — Extracted DNA concentration per sample measured by fluorometer. [file peerj-04-1913-s001.docx]

| **Sample** | **Extracted DNA (ng/μl)** |
| --- | --- |
| eel932BC1 | BD |
| eel932BC2 | BD |
| eel932BC3 | BD |
| Seep1a1441BC1 | BD |
| Seep1a1441BC2 | BD |
| DSS658BC1 | BD |
| DSS658BC2 | BD |
| DSS658BC3 | BD |
| Delta495aBC1 | BD |
| Delta495aBC2 | 0.05 |
| FixedBulk1 | 0.52 |
| FixedBulk2 | 0.55 |
| FixedBulk3 | 0.70 |
| UnfixedBulk2 | 0.80 |
| UnfixedBulk3 | 1.34 |
