# Supplementary material for: Characterization of microbial associations with methanotrophic archaea and sulfate-reducing bacteria through statistical comparison of nested Magneto-FISH enrichments
Source: PeerJ. 2016 Apr 18;4:e1913. doi: 10.7717/peerj.1913 (PMC4841229; doi:10.7717/peerj.1913)
Supplement: Table S2 — Expected and recovered sequence abundances among the mock communities show differential taxonomic biases. Fold Change is calculated by dividing the experimentally recovered relative abundance by the expected relative abundance. Four mock communities were designed with a selection of common methane seep bacterial and archaeal taxa at different relative abundance ratios. Mock community analysis revealed that relative abundances of Helicobacteraceae (Sulfurovum), Desulfobacteraceae (Seep-SRB1) and Desulfobulbaceae (Desulfobulbus) had little amplification bias as compared to other mock community taxa (fold change ranges 0.93–1.42, where 1.00 means expected relative abundance was returned). ANME-1b plasmids were also overall well represented (fold change 0.64 to 1.42) across the range of expected relative abundances (1% to 20%). In contrast, ANME-2a/b and ANME-2c plasmids were always under amplified in all of the mock communities (fold change 0.32 to 0.81). These results do not appear to correlate to primer hits in the SILVA SSU r123 database, where 89.5% of ANME-2c sequences were hit by 515f and 87.1% by 806r, but 94.3% of ANME-2a/b were hit by 515f and 806r. ANME-2a/b was a better match to the EMP primers, but both taxa were under amplified in mock community analysis. Amplification bias was not always uniform, where some templates saw varied amplification response depending on initial relative abundance in the mock community. The ANME-1a plasmid was over-amplified (3.35–2.44 fold change; Table S2) when the plasmid was at 5% relative abundance and lower (Mock Communities 1–3). However, Mock Community 4 with the highest relative abundance (20%) of ANME-1a plasmids, saw templates amplified to the expected relative abundance (0.97 fold change). Thaumarchaeota: miscellaneous Crenarchaeota Group followed a similar pattern to ANME-1a: where it was 1% expected relative abundance, the fold change is ∼5, and where it was 10% expected relative abundance, the fold change was less [file peerj-04-1913-s002.docx]

| **Plasmid Taxonomy** |  | **Mock 1** | **Mock 2** | **Mock 3** | **Mock 4** |
| --- | --- | --- | --- | --- | --- |
| ***Desulfobulbaceae* (DSB)** | *Expected* | ***3.0%*** | ***3.0%*** | ***11.0%*** | ***11.0%*** |
|  | Experimental | 3.0% | 3.2% | 11.6% | 12.9% |
|  | **Fold Change** | **0.99** | **1.05** | **1.05** | **1.17** |
|  | Std. Dev. | 0.06 | | 0.10 | |
| ***Helicobacteraceae*** | *Expected* | ***25.0%*** | ***25.0%*** | ***1.0%*** | ***1.0%*** |
|  | Experimental | 28.0% | 23.4% | 1.4% | 1.2% |
|  | **Fold Change** | **1.12** | **0.93** | **1.38** | **1.17** |
|  | Std. Dev. | 0.18 | | 0.16 | |
| ***Desulfobacteraceae* (DSS)** | *Expected* | ***9.0%*** | ***9.0%*** | ***31.0%*** | ***31.0%*** |
|  | Experimental | 12.7% | 11.0% | 34.8% | 31.5% |
|  | **Fold Change** | **1.42** | **1.22** | **1.12** | **1.02** |
|  | Std. Dev. | 0.15 | | 0.10 | |
| **ANME-1a** | *Expected* | *1.0%* | *4.0%* | *5.0%* | *20.0%* |
|  | Experimental | 3.4% | 9.9% | 14.5% | 19.4% |
|  | **Fold Change** | **3.35** | **2.48** | **2.90** | **0.97** |
| **ANME-1b** | *Expected* | ***1.0%*** | ***4.0%*** | ***5.0%*** | ***20.0%*** |
|  | Experimental | 1.4% | 3.4% | 6.9% | 12.8% |
|  | **Fold Change** | **1.42** | **0.85** | **1.37** | **0.64** |
| **ANME-2a/b** | *Expected* | ***6.0%*** | ***2.0%*** | ***30.0%*** | ***10.0%*** |
|  | Experimental | 3.7% | 0.6% | 10.2% | 6.2% |
|  | **Fold Change** | **0.61** | **0.32** | **0.34** | **0.62** |
| **ANME-2c** | *Expected* | ***3.5%*** | ***1.0%*** | ***15.0%*** | ***5.0%*** |
|  | Experimental | 1.9% | 0.5% | 6.9% | 4.1% |
|  | **Fold Change** | **0.55** | **0.49** | **0.46** | **0.81** |
| **Miscellaneous Crenarchaeota Group** | *Expected* | ***10.0%*** | ***10.0%*** | ***1.0%*** | ***1.0%*** |
|  | Experimental | 14.8% | 14.9% | 5.9% | 4.7% |
|  | **Fold Change** | **1.48** | **1.49** | **5.87** | **4.69** |
|  | Std. Dev. | 0.01 | | 0.22 | |
| ***Thermoplasmatales*** | *Expected* | ***41.5%*** | ***42.0%*** | ***1.0%*** | ***1.0%*** |
|  | Experimental | 30.8% | 30.7% | 6.5% | 6.1% |
|  | **Fold Change** | **0.74** | **0.73** | **6.48** | **6.09** |
|  | Std. Dev. | 0.01 | | 0.06 | |
