# Supplementary material for: Characterization of microbial associations with methanotrophic archaea and sulfate-reducing bacteria through statistical comparison of nested Magneto-FISH enrichments
Source: PeerJ. 2016 Apr 18;4:e1913. doi: 10.7717/peerj.1913 (PMC4841229; doi:10.7717/peerj.1913)
Supplement: Table S3 — Total sequences per sample after mothur processing and 0.1% bulk sediment cutoff and total sequences remaining for the most abundant ANME-2c, SEEP-SRB1, and ANME-1a OTUs. We also performed a BLASTN (Maddenm 2002) search of all contigs from all samples against an in-house database of 155 ANME-2c 16S rRNA sequences of >500 bp. This yielded 1,395 iTag sequences with an e-value greater than or equal to 10−130, corresponding to 99–100% sequence identity match to sequences from our ANME-2c database. We then tracked this set of BLAST match contigs through each step in the mothur pipeline, with a final result of 1,260 sequences remaining in this BLAST set from the original contig file. Thus 92% of our ANME-2c BLAST hit set remained through the mothur processing pipeline. This suggests that the lack of ANME-2c sequences in our downstream database was not due to spurious removal during sequence processing. [file peerj-04-1913-s003.docx]

|  | after 0.1% removal | ANME-2c | SEEP-SRB1 | ANME-1a |
| --- | --- | --- | --- | --- |
| Sample ID | Sequences | Otu16818 | Otu17765 | Otu12964 |
| ETR-D1-DSS658BC1 | 3460 | 0 | 120 | 67 |
| ETR-D2-DSS658BC2 | 5310 | 0 | 193 | 78 |
| ETR-D3-DSS658BC3 | 9859 | 0 | 393 | 804 |
| ETR-E1-eel932BC1 | 6304 | 0 | 279 | 36 |
| ETR-E2-eel932BC2 | 5253 | 0 | 100 | 176 |
| ETR-E3-eel932BC3 | 3293 | 0 | 414 | 52 |
| ETR-F1-FixedBulk1 | 12257 | 21 | 171 | 1263 |
| ETR-F2-FixedBulk2 | 4957 | 63 | 70 | 596 |
| ETR-F3-FixedBulk3 | 14400 | 27 | 215 | 1192 |
| ETR-S1-Seep1a1441BC1 | 2562 | 0 | 200 | 64 |
| ETR-S2-Seep1a1441BC2 | 7867 | 0 | 408 | 865 |
| ETR-U2-UnfixedBulk2 | 14441 | 54 | 309 | 1124 |
| ETR-U3-UnfixedBulk3 | 11447 | 32 | 244 | 1180 |
| ETR-delta1-Delta495aBC1 | 1552 | 0 | 0 | 0 |
| ETR-delta2-Delta495aBC2 | 3790 | 0 | 91 | 0 |
| Total | 171154 | 197 | 3207 | 7497 |
| Percent of Unfixed Bulk Sediment | - | 0.33% | 2.14% | 8.90% |
