# Supplementary material for: Characterization of microbial associations with methanotrophic archaea and sulfate-reducing bacteria through statistical comparison of nested Magneto-FISH enrichments
Source: PeerJ. 2016 Apr 18;4:e1913. doi: 10.7717/peerj.1913 (PMC4841229; doi:10.7717/peerj.1913)
Supplement: Table S4 — Mock Community sequencing error rates (0.025–0.095%; Table S4) were of the same magnitude as Kozich et al. (∼0.01%, 2013). Rarefaction of the mock community to 5,000 sequences shows OTU inflation rates of 3 to 4 times expected number of OTUs, after 97% OTU clustering and removal of singletons. The inflation rate is calculated by total number of OTUs recovered divided by original number of template plasmids. Since our environmental mock community only had 12 templates, the number of spurious OTUs is expected to be high (Huse et al., 2010). Experimental sediment samples have 10 to 100 times more templates, so inflation rates are expected to be much lower (10–1%). [file peerj-04-1913-s004.docx]

| **Sample** | **OTU@5000** | **OTU@5000, singletons remv.** | **OTU@5000 singletons remv. inflation rate** | **Error Rate** |
| --- | --- | --- | --- | --- |
| Mock 1 | 65 (57-73) | 32 (28-36) | 2.7 | 0.025% |
| Mock 2 | 58 (50-68) | 30 (26-34) | 2.5 | 0.033% |
| Mock 3 | 113 (102-123) | 38 (35-40) | 3.2 | 0.095% |
| Mock 4 | 85 (77-94) | 38 (35-41) | 3.2 | 0.085% |
