# Supplementary material for: Characterization of microbial associations with methanotrophic archaea and sulfate-reducing bacteria through statistical comparison of nested Magneto-FISH enrichments
Source: PeerJ. 2016 Apr 18;4:e1913. doi: 10.7717/peerj.1913 (PMC4841229; doi:10.7717/peerj.1913)
Supplement: Table S5 — All associations that occurred in at least 50 out of 100 networks for combined Magneto-FISH and bulk sediment samples with their number of occurrences, average correlation, and p-values. [file peerj-04-1913-s005.docx]

| **OTU_1** | **Tax_1** | **OTU_2** | **Tax_2** | **Occurrence** | **Correlation** | **Pvalue** |
| --- | --- | --- | --- | --- | --- | --- |
| Otu072 | ANME-1b | Otu090 | JS1 | 100 | 0.746 | 0.002 |
| Otu069 | OD1 | Otu077 | Pelobacter | 100 | 0.872 | 0 |
| Otu014 | AKAU3564_sed. | Otu082 | SEEP-SRB1 | 100 | 0.729 | 0.001 |
| Otu071 | Hyd24-12 | Otu083 | SEEP-SRB1 | 100 | 0.765 | 0 |
| Otu073 | Sulfurovum | Otu087 | Sulfurovum | 100 | 0.798 | 0.001 |
| Otu134 | MBG-B | Otu135 | OM1 | 99 | 0.737 | 0.001 |
| Otu123 | VC2.1_Bac22 | Otu128 | WS3 | 98 | 0.718 | 0.001 |
| Otu106 | OD1 | Otu115 | Desulfocapsa | 97 | 0.705 | 0.002 |
| Otu071 | Hyd24-12 | Otu097 | Spirochaeta | 96 | 0.708 | 0.002 |
| Otu083 | SEEP-SRB1 | Otu097 | Spirochaeta | 96 | 0.691 | 0.001 |
| Otu100 | OD1 | Otu107 | Anaerolineaceae | 95 | 0.709 | 0.001 |
| Otu021 | WF1B-44 | Otu022 | Anaerolineaceae | 95 | 0.681 | 0.001 |
| Otu068 | Desulfoluna | Otu119 | VC2.1_Bac22 | 95 | 0.692 | 0.001 |
| Otu077 | Pelobacter | Otu094 | BD2-2 | 94 | 0.703 | 0.002 |
| Otu073 | Sulfurovum | Otu101 | SEEP-SRB1 | 94 | 0.778 | 0.003 |
| Otu097 | Spirochaeta | Otu131 | Actibacter | 93 | -0.688 | 0.002 |
| Otu014 | AKAU3564_sed. | Otu089 | SEEP-SRB1 | 93 | 0.691 | 0.002 |
| Otu122 | SEEP-SRB4 | Otu131 | Actibacter | 92 | 0.679 | 0.002 |
| Otu112 | SEEP-SRB1 | Otu114 | JS1 | 92 | 0.663 | 0.003 |
| Otu023 | Anacalomicrobium | Otu024 | MSBL8 | 92 | 0.684 | 0.001 |
| Otu102 | SEEP-SRB1 | Otu116 | VC2.1_Bac22 | 92 | 0.701 | 0.001 |
| Otu098 | Desulfobulbus | Otu108 | BD2-2 | 91 | -0.668 | 0.003 |
| Otu069 | OD1 | Otu086 | OM1 | 91 | 0.688 | 0.003 |
| Otu090 | JS1 | Otu114 | JS1 | 90 | 0.685 | 0.002 |
| Otu095 | SEEP-SRB2 | Otu119 | VC2.1_Bac22 | 90 | 0.687 | 0.001 |
| Otu069 | OD1 | Otu094 | BD2-2 | 89 | 0.683 | 0.002 |
| Otu091 | SEEP-SRB2 | Otu114 | JS1 | 89 | 0.664 | 0.004 |
| Otu082 | SEEP-SRB1 | Otu089 | SEEP-SRB1 | 89 | 0.69 | 0.002 |
| Otu071 | Hyd24-12 | Otu100 | OD1 | 86 | 0.679 | 0.003 |
| Otu091 | SEEP-SRB2 | Otu107 | Anaerolineaceae | 85 | 0.688 | 0.003 |
| Otu087 | Sulfurovum | Otu099 | Sulfurovum | 85 | 0.675 | 0.004 |
| Otu094 | BD2-2 | Otu100 | MBG-B | 84 | 0.662 | 0.003 |
| Otu081 | Caldthrix | Otu086 | OM1 | 84 | -0.642 | 0.003 |
| Otu127 | Anaerolineaceae | Otu132 | Thiohalobacter | 84 | 0.651 | 0.002 |
| Otu098 | Desulfobulbus | Otu122 | SEEP-SRB4 | 81 | 0.66 | 0.003 |
| Otu091 | SEEP-SRB2 | Otu134 | MBG-B | 80 | 0.663 | 0.003 |
| Otu105 | CS-B046 | Otu108 | BD2-2 | 79 | 0.656 | 0.003 |
| Otu077 | Pelobacter | Otu086 | OM1 | 79 | 0.655 | 0.003 |
| Otu073 | Sulfurovum | Otu098 | Desulfobulbus | 78 | 0.677 | 0.004 |
| Otu016 | Anaerolineaceae | Otu092 | SB-5 | 77 | 0.649 | 0.003 |
| Otu068 | Desulfoluna | Otu095 | SEEP-SRB2 | 77 | 0.658 | 0.002 |
| Otu103 | ANME-2a/b | Otu135 | OM1 | 71 | 0.647 | 0.004 |
| Otu126 | WS3 | Otu129 | Clostridia | 70 | 0.653 | 0.003 |
| Otu090 | JS1 | Otu100 | OD1 | 69 | 0.647 | 0.004 |
| Otu122 | SEEP-SRB4 | Otu128 | WS3 | 68 | 0.641 | 0.004 |
| Otu027 | Anaerolineaceae | Otu030 | WCHB1-69 | 66 | 0.657 | 0.001 |
| Otu112 | SEEP-SRB1 | Otu121 | Desulfarc._unclt. | 65 | -0.648 | 0.004 |
| Otu084 | Desulfobulbus | Otu120 | Desulfococcus | 65 | 0.663 | 0.006 |
| Otu051 | Lutibacter | Otu096 | ANME-1b | 64 | -0.636 | 0.004 |
| Otu005 | Desulfocapsa | Otu032 | Hyd24-12 | 64 | 0.648 | 0.003 |
| Otu011 | Anaerolineaceae | Otu072 | ANME-1b | 63 | -0.639 | 0.003 |
| Otu072 | ANME-1b | Otu100 | OD1 | 59 | 0.637 | 0.005 |
| Otu072 | ANME-1b | Otu094 | BD2-2 | 58 | 0.646 | 0.003 |
| Otu124 | WS3 | Otu134 | MBG-B | 57 | 0.631 | 0.003 |
| Otu020 | pMC2A209 | Otu051 | Lutibacter | 55 | 0.646 | 0.003 |
| Otu113 | Desulfobulbus | Otu118 | SAR406 | 55 | 0.643 | 0.003 |
| Otu036 | Desulfocapsa | Otu046 | Sulfurimonas | 54 | 0.646 | 0 |
| Otu069 | OD1 | Otu081 | Caldthrix | 52 | -0.633 | 0.004 |
| Otu108 | BD2-2 | Otu120 | Desulfococcus | 50 | 0.632 | 0.003 |
| Otu103 | ANME-2a/b | Otu122 | SEEP-SRB4 | 50 | 0.635 | 0.004 |
| Otu046 | Sulfurimonas | Otu054 | WCHB1-69 | 50 | 0.636 | 0.001 |
