# Supplementary material for: Characterization of microbial associations with methanotrophic archaea and sulfate-reducing bacteria through statistical comparison of nested Magneto-FISH enrichments
Source: PeerJ. 2016 Apr 18;4:e1913. doi: 10.7717/peerj.1913 (PMC4841229; doi:10.7717/peerj.1913)
Supplement: Figure S1 [file peerj-04-1913-s006.pdf]

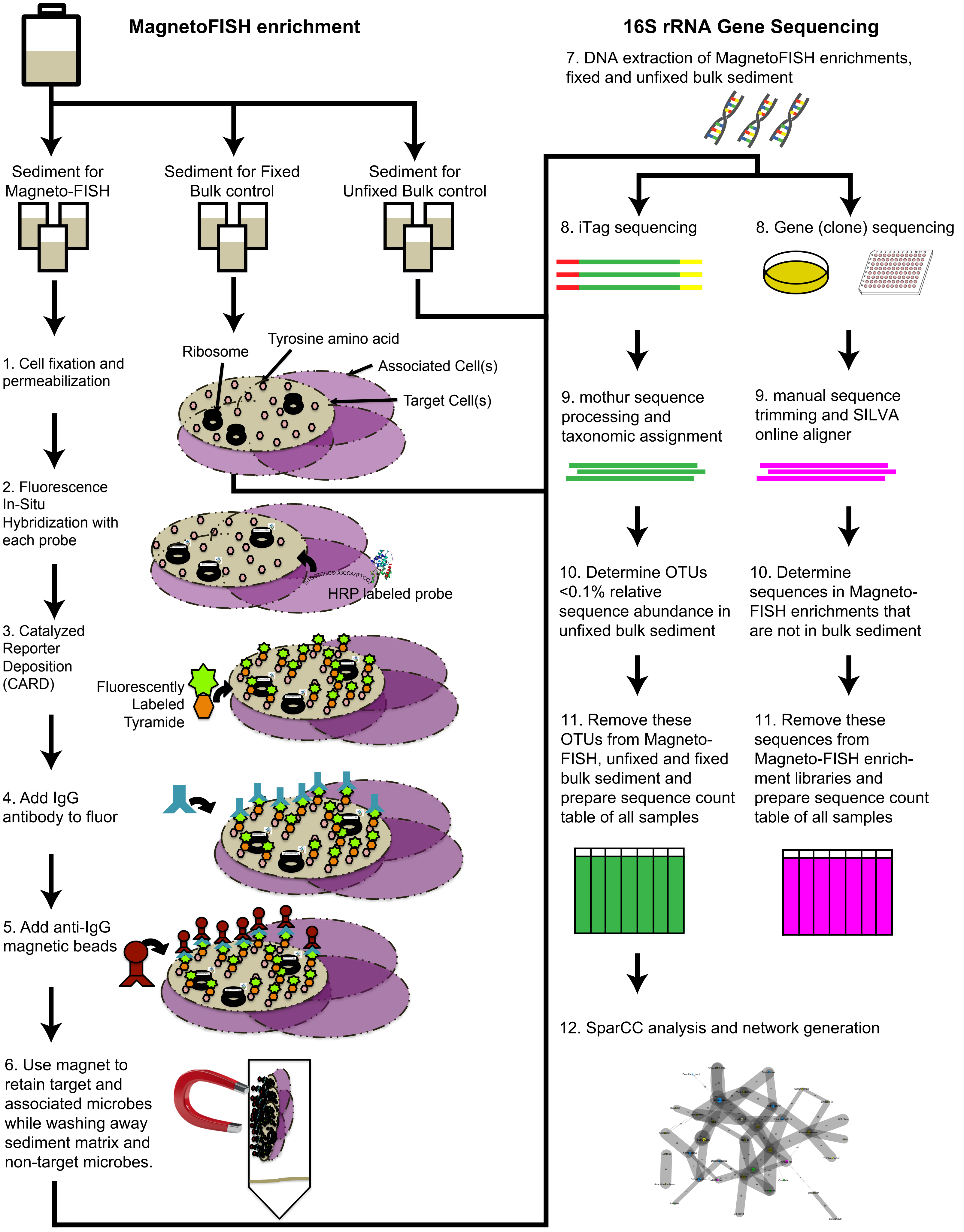
MagnetoFISH enrichment
16S rRNA Gene Sequencing
7. DNA extraction of MagnetoFISH enrichments, fixed and unfixed bulk sediment
Sediment for Magneto-FISH
Sediment for Fixed Bulk control
Sediment for Unfixed Bulk control
8. iTag sequencing
8. Gene (clone) sequencing
1. Cell fixation and permeabilization
Ribosome
Tyrosine amino acid
Associated Cell(s)
Target Cell(s)
9. mothur sequence processing and taxonomic assignment
9. manual sequence trimming and SILVA online aligner
2. Fluorescence In-Situ Hybridization with each probe
HRP labeled probe
10. Determine OTUs <0.1% relative sequence abundance in unfixed bulk sediment
10. Determine sequences in Magneto-FISH enrichments that are not in bulk sediment
3. Catalyzed Reporter Deposition (CARD)
Fluorescently Labeled Tyramide
11. Remove these OTUs from Magneto-FISH, unfixed and fixed bulk sediment and prepare sequence count table of all samples
11. Remove these sequences from Magneto-FISH enrichment libraries and prepare sequence count table of all samples
4. Add IgG antibody to fluor
5. Add anti-IgG magnetic beads
12. SparCC analysis and network generation
6. Use magnet to retain target and associated microbes while washing away sediment matrix and non-target microbes.
