# Supplementary material for: Characterization of microbial associations with methanotrophic archaea and sulfate-reducing bacteria through statistical comparison of nested Magneto-FISH enrichments
Source: PeerJ. 2016 Apr 18;4:e1913. doi: 10.7717/peerj.1913 (PMC4841229; doi:10.7717/peerj.1913)

Functional Gene	dsrA	aprA	soxB
Desulfobacteraceae (DSB)	15	7	
Desulfobulbaceae (DBB)	1	0	
Gammaproteobacteria (Endosymbiont)		5	
Other (Cluster B)		1	
Epsilonproteobacteria (Sulfurimonas)			20
Epsilonproteobacteria (Sulfurovum)			2

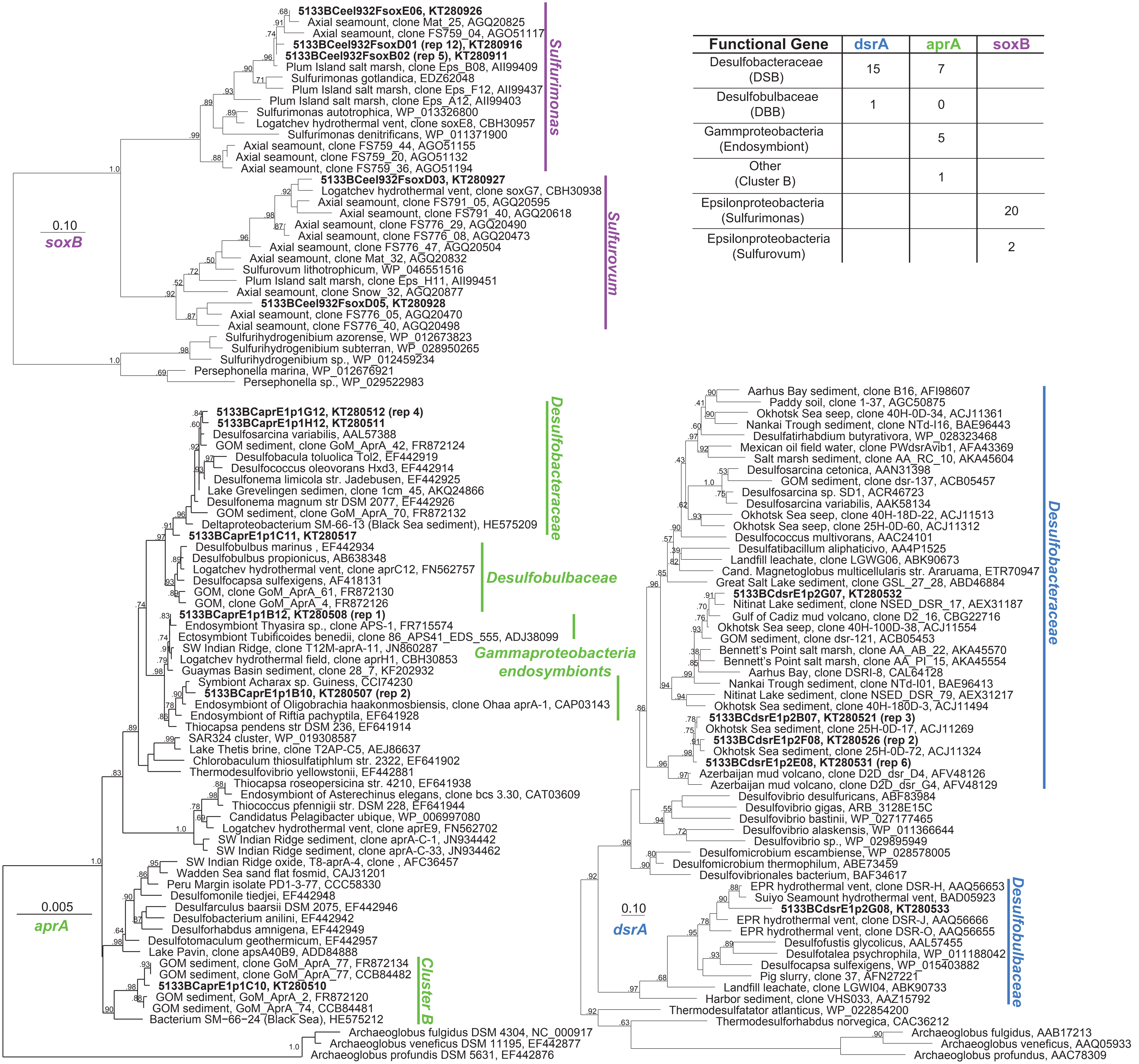

Supplement: Figure S2 — Phylogenetic trees of SoxB, AprA, and DsrA functional genes from a MSMX-Eel_932 Magneto-FISH enrichment. aLRT SH-like values above 50% displayed for branch support. Similar sequence clusters represented by one sequence are indicated in parentheticals, and sequences from this study are in bold. Clones recovered are also summarized in table form. As another method of assessing Magneto-FISH diversity, we examined functional genes relating to the sulfur cycle. This method can also provide insight into phylogenetic connections between 16S rRNA and sulfur cycling functional genes. Clone libraries were constructed from an MSMX-Eel_932 Magneto-FISH capture and performed with sediment as iTag libraries (see ‘Materials & Methods’). The following genes relating to sulfur cycling pathways were chosen for this analysis: soxB (sulfur oxidation, protein-S-thiocysteine sulfate hydrolase), aprA (sulfur oxidation and reduction, adenylylsulfate reductase α subunit), and dsrA (sulfur oxidation and reduction, dissimilatory sulfite reductase). Phylogenetic analysis of soxB clones from the MSMX-Eel_932 Magneto-FISH returned only Epsilonproteobacteria sequences from both Sulfurovum (2 clones) and Sulfurimonas (20 clones) clades (Fig. S1). From a total of 13 aprA clones, 7 were retrieved from the Desulfobacteraceae clade (SEEP-SRB1 containing), none from the Desulfobulbaceae, 5 from Gammaproteobacteria Endosymbiont clade, and 1 from the “Cluster B” GoM clone clade (Meyer & Kuever, 2007). 15 of 16 dsrA clones were from the Desulfobacteraceae clade, with one clone from the Desulfobulbaceae clade (Müller et al., 2015). Functional gene clone libraries were not only successful in providing another means to assess Magneto-FISH enrichment, but provide an example of how this technique can be utilized to target specific 16S rRNA populations and the metabolic diversity contained. This is particularly useful in cases where 16S rRNA and functional gene phylogenies are not well aligned. [file peerj-04-1913-s007.pdf]
